# Metabolite Profiling of Pig Seminal Plasma Identifies Potential Biomarkers for Sperm Resilience to Liquid Preservation

**DOI:** 10.3389/fcell.2021.669974

**Published:** 2021-05-28

**Authors:** Yentel Mateo-Otero, Pol Fernández-López, Jordi Ribas-Maynou, Jordi Roca, Jordi Miró, Marc Yeste, Isabel Barranco

**Affiliations:** ^1^Biotechnology of Animal and Human Reproduction (TechnoSperm), Institute of Food and Agricultural Technology, University of Girona, Girona, Spain; ^2^Unit of Cell Biology, Department of Biology, Faculty of Sciences, University of Girona, Girona, Spain; ^3^Centre d’Estudis Avançats de Blanes (CEAB), Spanish Research Council (CSIC), Girona, Spain; ^4^Department of Animal Medicine and Surgery, Faculty of Veterinary Medicine, University of Murcia, Murcia, Spain; ^5^Equine Reproduction Service, Department of Animal Medicine and Surgery, Faculty of Veterinary Medicine, Autonomous University of Barcelona, Barcelona, Spain; ^6^Department of Veterinary Medical Sciences, Via Tolara di Sopra, Bologna, Italy

**Keywords:** metabolite, metabolomic, seminal plasma, liquid storage, sperm quality, biomarkers, pig

## Abstract

Metabolomic approaches allow the study of downstream gene expression events since metabolites are considered as the products of cell signaling pathways. For this reason, many studies in humans have already been conducted to determine the influence of the metabolites present in seminal plasma (SP) on sperm physiology, and to identify putative biomarkers. However, in livestock species, these relationships are yet to be uncovered. Thus, the present study aimed to explore: (i) if concentrations of metabolites in pig SP are related to sperm quality and functionality, and (ii) if they could predict the sperm resilience to liquid storage at 17°C. To this end, 28 ejaculates were individually collected and split into three aliquots: one was used for SP analysis through nuclear magnetic resonance (NMR) spectroscopy; another served for the evaluation of sperm concentration and morphology; and the last one was utilized to determine sperm functionality parameters using computer-assisted sperm analysis (CASA) and flow cytometry after 0 h and 72 h of liquid-storage at 17°C. NMR analysis allowed the identification and quantification of 23 metabolites present in pig SP which, except for fumarate, were not observed to follow a breed-dependent behavior. Moreover, specific relationships between metabolites and sperm variables were identified: (i) glutamate, methanol, trimethylamine N-oxide, carnitine, and isoleucine were seen to be related to some sperm quality and functionality parameters evaluated immediately after semen collection; (ii) leucine, hypotaurine, carnitine and isoleucine were found to be associated to the sperm ability to withstand liquid storage; and (iii) Bayesian multiple regression models allowed the identification of metabolite patterns for specific sperm parameters at both 0 h and 72 h. The identification of these relationships opens up the possibility of further investigating these metabolites as potential sperm functional biomarkers.

## Introduction

Artificial insemination (AI) remains the most valuable and efficient reproductive biotechnology used for pig breeding, gathering more than 90% of cases in countries with intensive pork production (Roca et al., 2016; Yeste, 2017; Waberski et al., 2019). In spite of the advances performed in cryopreservation procedures, liquid storage at 17 °C continues to be the most useful and preferable biotechnology for preserving pig seminal AI-doses until insemination is carried out (Kumaresan et al., 2009; Waberski et al., 2019). During liquid-storage of seminal AI-doses, sperm undergo injuries, leading to a decline in sperm quality, functionality and fertilizing ability within the first 72 h of storage (Waberski et al., 2011; Yeste, 2017). One of the main concerns is related to differences in the sperm ability to withstand liquid-storage between pig seminal AI-doses (Parrilla et al., 2012; Zakošek Pipan et al., 2014; Roca et al., 2015). This situation evidences that current sperm assessments used for ejaculate selection (which include sperm quantity, motility, and morphology) are not accurate enough to predict sperm fertilizing capacity after liquid preservation (Roca et al., 2016; Waberski et al., 2019). This limitation undermines the ability to face new challenges in swine industry, as implementing new AI-procedures (such as a reduction in sperm numbers/AI-dose) may lead to a decrease on *in vivo* fertility outcomes (Roca et al., 2016). In the last years, many efforts have been aimed at identifying biomarkers of sperm resilience to liquid preservation in seminal plasma (SP) (Barranco et al., 2015a,b, 2016, 2019, 2020; Recuero et al., 2019; Zakošek Pipan et al., 2014). The heterogeneous and complex composition of SP, together with the fact that this fluid is able to interact with sperm, make it a valuable source of potential biomarkers (Rodríguez-Martínez et al., 2011).

Over the last decade, -omics technologies (including proteomics, transcriptomics, metabolomics…) have emerged as powerful tools to uncover the molecular factors and regulatory networks involved in reproductive events, thus providing a worthwhile and broad strategy for the study of these multifactorial processes (Egea et al., 2014). Metabolomics employs spectral and analytical approaches to provide a large-scale identification of metabolites, which are the end-products of downstream events of gene expression (Goodacre et al., 2004). Hence, this technique leads to a more realistic approach of cellular physiological activities compared with other -omics, such as transcriptomics or proteomics (Singh and Sinclair, 2007). In the last few years, the study of metabolites as markers of patho- and physiological reproductive processes, in both males and females, has gained much relevance (Egea et al., 2014; Uyar and Seli, 2014; Minai-Tehrani et al., 2016; Nobakht and Gh, 2018; Darbandi et al., 2019; Asampille et al., 2020). Focusing on SP, several studies performed in humans reported that certain SP-metabolites are involved on sperm physiology and fertility disorders (Bonechi et al., 2015; Engel et al., 2019; Wang et al., 2019; Mumcu et al., 2020; Xu et al., 2020). In livestock species, only few reports conducted in cattle pointed out specific SP-metabolites as *in vivo* fertility biomarkers (Kumar et al., 2015; Cazaux Velho et al., 2018).

In a previous study, we described, for the first time, the pig SP metabolomic profile and we suggested that certain SP-metabolites could play a key role in reproductive processes (Mateo-Otero et al., 2020). As far as we know, there are no data about the relationship between SP-metabolites and the sperm ability to withstand liquid preservation in any species. Therefore, an exploratory approach was carried out to evaluate the putative relationship between sperm quality/functionality parameters of liquid-stored semen samples and SP-metabolites identified using nuclear magnetic resonance (NMR) spectroscopy. Specifically, the present study aimed to explore: (i) whether the concentration/presence of SP-metabolites in ejaculates collected from AI-boars is related with sperm quality/functionality parameters, and (ii) if such SP-metabolites could predict the resilience of seminal AI-doses to liquid preservation at 17°C. In this regard, these SP-metabolites may be of practical relevance for a better assessment of pig semen quality, contributing to the successful improvement of AI-efficiency.

## Materials and Methods

### Reagents

Except as otherwise indicated, reagents were purchased from Merck KGaA (Darmstadt, Germany) and fluorochromes for sperm analysis from Thermo Fisher Scientific (Waltham, MA, USA). The commercial extender used to dilute semen samples was Biosem + (Magapor, Zaragoza, Spain).

### Experimental Design

Twenty-eight entire ejaculates were individually collected from 28 healthy and fertile AI-boars (one ejaculate per boar) from different breeds (Pietrain, Duroc, Landrace and Tempo). The entire ejaculates were split into three aliquots. The first one was used for SP-harvesting, on which the metabolomic analysis was performed. The second one was utilized for sperm concentration and morphology assessments. The third aliquot was extended alike an AI-dose (30 × 10^6^ sperm/mL in Biosem +) and stored up to 72 h at 17°C (FOC 120E Cooled Incubator; VELP Scientifica, Usmate, Italy). Sperm quality and functionality parameters were assessed in this latter aliquot at different time-points (0 and 72 h), in terms of sperm motility, viability, acrosome damage, intracellular hydrogen peroxide (H_2_O_2_) generation by viable sperm, and membrane lipid disorder in viable sperm.

### Boars and Ejaculates

Ejaculates used in the present study were supplied by a Spanish AI-Centre (AIM Ibérica, Topigs Norsvin Spain; Calasparra, Murcia, Spain). This AI-Centre complies with Spanish (ES300130640127, August 2006) and European (ES13RS04P, July 2012) guidelines for animal health and welfare, and pig ejaculate collection and commercialization of AI-doses. As semen samples were provided by that AI-Centre and authors did not manipulate any animal, no ethical permission was needed.

All entire ejaculates were collected through a semi-automatic collection system (Collectist^®^, IMV Technologies, L’Aigle, France) from sexually mature (1 to 3 years old), fertile boars that were subjected to routine collection (twice per week) for elaborating commercial AI-doses. These AI-boars were housed in individual pens with controlled environment temperature (15–25°C) and exposed to a total of 16 h of light (natural plus artificial). They were fed with commercial feedstuff consistent with the nutritional necessities of AI-boars, and were provided with water *ad libitum*. All ejaculates collected for this study fulfilled the sperm quality thresholds for commercial AI-doses (namely a minimum sperm concentration of 200 × 10^6^ sperm/mL and more than 70% of motile sperm and 75% of sperm with normal morphology).

### Seminal Plasma Processing and Storage

For SP-harvesting, ejaculates were subjected to double-centrifugation right after collection (1,500 × *g* for 10 min at room temperature Rotofix 32A, Hettich Centrifuge United Kingdom, Newport Pagnell, Buckinghamshire, England, United Kingdom). Subsequently, SP samples (second supernatants) were microscopically analyzed (Eclipse E400; Nikon, Tokyo, Japan) to warrant sperm absence. Finally, SP-samples were stored in cryotubes at −80°C (Ultra Low Freezer; Haier Inc., Qingdao, China) until metabolomic analysis was carried out, when they were thawed on ice.

### Assessment of Sperm Quality and Functionality Parameters

Eight sperm quantity and functionality parameters were assessed in each semen sample, specifically: (i) sperm concentration, (ii) total sperm count, (iii) sperm morphology, (iv) sperm motility, (v) sperm plasma membrane integrity (viability), (vi) acrosome damage in viable sperm, (vii) intracellular H_2_O_2_ generation by viable sperm, and (viii) plasma membrane lipid disorder of viable sperm.

Sperm concentration was assessed by a high-precision automated cell counter (NucleoCounter^®^, NC-100^TM^, ChemoMetec, Allerod, Denmark). Then, the total sperm number of each ejaculate was calculated by multiplying sperm concentration per ejaculate volume (assessed in a graduated tube).

For sperm morphology assessment, semen samples were fixed with 0.12% formaldehyde saline solution (Panreac, Barcelona, Spain) and examined under a phase-contrast microscope at 1,000 × magnification (Nikon Labophot, Nikon, Tokio, Japan). A total of 200 sperm per sample were counted and classified as: (i) morphologically normal, (ii) with abnormal head, (iii) with acrosome defects, (iv) with proximal cytoplasmic droplets, (v) with distal cytoplasmic droplets, (vi) with folded tails, and (vii) with coiled tails.

Sperm motility was evaluated using a computer-assisted sperm analyzer (CASA, ISASV1^®^, Proiser R + D S.L., Paterna, Spain). With this purpose, 5 μL of each semen sample (30 × 10^6^ sperm/mL in Biosem +) were pipetted onto a Makler chamber (Sefi Medical Instruments, Haifa, Israel), previously warmed to 38°C. In each semen sample, a total of ten different fields, gathering more than 600 sperm, were acquired and examined under an Olympus BX41 microscope (Olympus, Tokyo, Japan) with a negative phase-contrast field (Olympus 10 × 0.30 PLAN objective; Olympus). Percentages of sperm that showed an average path velocity ≥ 20 μm/s (total motile sperm) and that exhibited a rapid and progressive movement with a straight-line velocity ≥ 40 μm/s (progressive motile sperm) were recorded.

Sperm viability, acrosome damage, intracellular H_2_O_2_ production and membrane lipid disorder were assessed with a flow cytometer (BD FACS Canto II; Becton Dickinson & Company, Franklin Lakes, NJ, USA). The optical configuration of this device included three detector arrays: one octagon, which detects light from 488 nm laser (blue), and two trigons, which detect light from 633 nm (red) and 405 nm (violet) lasers. Data were collected and compensated using BD FACSDiva Software (Becton Dickinson & Company). Events were triggered by forward scatter (FSC) and side scatter (SSC), and non-sperm particles were gated out based on Hoechst 33342 (H-42) fluorescence, which was detected using 450/50 nm band-pass (BD) filter. For each semen sample and sperm parameter, three technical replicates (with 10,000 [H-42]-positive events each one) were evaluated. All fluorescence parameters were log-transformed, whereas FSC and SSC signals were linearly processed. Unstained and single-stained samples were used for setting electronic volume (EV) gain and compensations. For all samples, laser voltage and flow rate were constant.

Sperm viability and acrosome damage were assessed by triple-staining with H-42, propidium iodide (PI) and fluorescein-conjugated peanut agglutinin (PNA-FITC). Fluorescence of PI and PNA-FITC was detected using 670 nm long-pass (LP) filter and 530/30 nm BP filter, respectively. With this purpose, 100 μL of each semen sample (30 × 10^6^ sperm/mL in Biosem +) were incubated with 3 μL H-42 (0.05 mg/mL in phosphate-buffered saline, PBS), 2 μL PI (0.5 mg/mL in PBS) and 2 μL PNA-FITC (100 μg/mL in PBS) for 10 min at 37°C (Sanyo MIR-153 incubator, Gemini BV, Apeldoorn, Netherlands). After this period, 400 μL PBS were added to each sample. Percentages of viable spermatozoa (H-42 + /PI-) with an intact (PNA-FITC-) and non-intact (PNA-FITC +) acrosome membrane were recorded.

To assess the intracellular H_2_O_2_ production by viable sperm, a triple-staining with H-42, PI, and 5- and 6-chloromethyl-2′,7′-dichlorodihydrofluorescein diacetate acetyl ester (CM-H_2_DCFDA) was performed. Fluorescence of CM-H_2_DCFDA was detected using 530/30 nm BP filter following excitation at 488 nm. Briefly, 50 μL of each semen sample (30 × 10^6^ sperm/mL in Biosem +) were incubated with 1.5 μL H-42 (0.05 mg/mL in PBS), 1 μL PI (0.5 mg/mL in PBS), and 1 μL CM-H_2_DCFDA (1 mM in dymetilsulfoxide, DMSO) in 950 μL of PBS at 37°C for 30 min. A semen sample incubated with all fluorochromes plus 1 μL of tert-butyl hydroperoxide solution (70% in distilled water) was used as a control. The percentage of viable sperm (H-42 + /PI-) that exhibited high intracellular H_2_O_2_ generation (2′,7′-dichlorofluorescein, DCF +) was recorded.

To evaluate membrane lipid disorder of viable sperm, a triple-staining with H-42, Yo-Pro-1, and Merocyanine 540 (M-540) was carried out. Fluorescence of M-540 and Yo-Pro-1 was detected using 585/42 nm and 530/30 nm BP filters, respectively. Fifty μL of each semen sample (30 × 10^6^ sperm/mL in Biosem +) were incubated with 2.5 μL H-42 and 10 μL Yo-Pro-1 (2.5 μM in DMSO) in 950 μL of PBS at 37°C for 8 min. After this period, 26 μL of M540 (0.1 mM in DMSO) were added to each sample and incubated at 37°C for further 2 min. The percentage of viable spermatozoa (H-42 + /Yo-Pro-1-) that exhibited high plasma membrane disorder (M-540 +) was recorded.

### Ejaculate Resilience to Liquid Preservation

The ability of each sample to withstand liquid preservation was evaluated as the loss of sperm quality after 72 h. With this purpose, the following formula was used: [(q1-q0)/q0] × 100, where q1 is the value of each sperm parameter after 72 h of liquid storage at 17°C, and q0 is the value of each sperm parameter evaluated immediately after ejaculate collection (0 h). This formula was applied to the following sperm parameters: (i) percentages of viable sperm, (ii) percentages of viable sperm with a non-intact acrosome, (iii) percentages of viable sperm with high intracellular H_2_O_2_ levels, (iv) percentages of viable sperm with high plasma membrane disorder, and (v) percentages of total motile sperm. Regarding progressive motility, proportions of progressively motile sperm at 0 h or 72 h were calculated relative to total motility as follows: (progressive motility)/(total motility) × 100.

### ^1^H RMN Analysis

A 500-μL aliquot of thawed SP-samples was vortexed, and centrifuged at 14,000 g and 4 °C for 90 min (Rotofix 32A, Hettich Centrifuge UK; Newport Pagnell Buckinghamshire, United Kingdom) using 0.5 mL-Amicon^®^ Ultra Centrifugal Filters to remove proteins and remaining cell debris. These filters were previously washed with PBS and prepared by centrifuging three times at 14,000 g and 4 °C for 20 min. Then, the eluted fraction was mixed with 100 μL PBS containing 10% D_2_O with 0.33% of DSS (Merck KgaA, Darmstadt, Germany; pH 7.4), and transferred into a 5-mm Wilmad^®^ NMR tube (Merck KgaA) to generate ^1^H NMR spectra.

NMR spectra were obtained using a Bruker 600-MHz AVANCE III NMR spectrometer (Bruker Biospin, Rheinstetten, Germany) operating at a ^1^H frequency of 600.13 MHz and a temperature of 300 °K with an equilibration period of 10 min. The ^1^D-^1^H-nuclear Overhauser effect spectroscopy (^1^D-NOESY) pulse sequence from the Bruker library was used (noesygppr1d). Relevant parameters were: mixing time: 100 ms (d8), recovery delay: 2 s (d1), 90° pulse: 10.39 μs (p1), spectral width: 7211.539 Hz, spectral size: 32 k, number of scans: 128, and acquisition time: 2.27 s.

### Data Processing and Analysis

Spectra were processed and analyzed with Chenomx 8.0 profiler. The software provides tools for automatic phase, baseline correction and reference calibration as well as metabolites libraries for profiling. Determination of concentration for each metabolite was calculated in relation to DSS concentration (0.216 mM).

### Statistical Analysis

All analysis were performed using R software (version 4.0.2)^1^. For all analysis, the level of significance was set at *P* ≤ 0.05. Statistical analysis of NMR data was performed in three steps. First, the breed effect on SP-metabolite concentration/presence was assessed performing a Wilcoxon rank sum test, which avoids the assumptions of normality and homoscedasticity as not all SP-metabolites fulfilled these requisites. The same test was also used to check whether age distribution between Duroc and Pietrain breeds differed.

Second, principal component analysis (PCA) was carried out to evaluate whether data could be categorized to few variables/components, as this avoided using all sperm parameters and made classifying ejaculates into two groups of high and low quality easier. As this could not be achieved, Pearson correlations were employed to quantify the relationship between single SP-metabolites and sperm parameters.

Third, multiple linear regression analyses were performed using a Bayesian framework with the functionality of the R package ‘rstanarm’ (R package version 2.21.1; Goodrich et al., 2020). All models were performed with a prior non-informative distribution, with the highest sensitivity and 4,000 iterations of sampling. The remaining sperm parameters were left by default. The Bayesian approach was chosen over the classical frequentist framework due to the nature of the dataset. Because linear regression models tend to be overfitted in datasets where the number of columns (variables or predictors) is similar to the number of rows (samples), Bayesian models allow quantifying the uncertainty of such models. In other words, it is possible to have a good sense of how much sensitive or precise these models are. This was performed for the two time-points (i.e., 0 h and 72 h) with the particularity that, for sperm resilience to liquid storage, logistic regression was used for modeling percentages of viable sperm with high intracellular H_2_O_2_ production and high plasma membrane destabilization. For these latter analyses, medians were used to discriminate the two quality groups: equal or below the median was considered as 0, and above the median was considered as 1. Finally, Pearson correlations were also calculated to determine inter-metabolite relationships.

## Results

### Metabolite Profile of Pig SP and Differences Between Breeds

**FIGURE 1:**
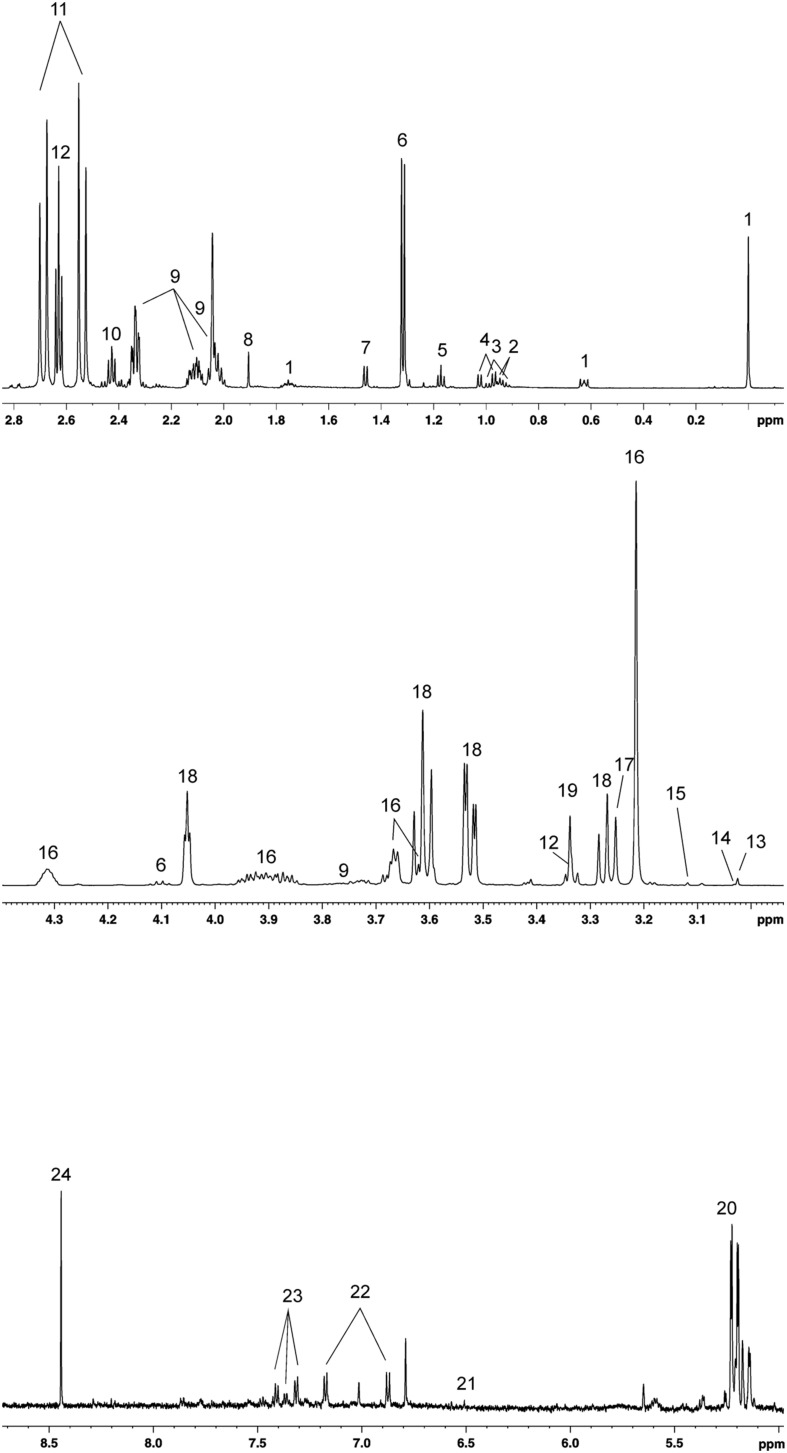
1H-NMR (noesygppr1d) profile (600MHz) from 0 to 8 ppm of pig seminal plasma. (1) DSS, (2) leucine, (3) isoleucine, (4) valine, (5) ethanol, (6) lactate, (7) alanine, (8) acetate, (9) glutamate, (10) carnitine, (11) citrate, (12) hypotaurine, (13) creatine, (14) creatine phosphate, (15) malonate, (16) sn-glycero-3-phosphocholine, (17) TMAO, (18) myo-inositol, (19) methanol, (20) glucose, (21) fumarate, (22) tyrosine, (23) phenylalanine, and (24) formate.

A total of 23 metabolites were identified and quantified in all SP-samples analyzed, observing that SP-metabolite concentrations varied between ejaculates (Table 1). The ^1^H-NMR (noesygppr1d) profile of pig SP measured at 600 MHz is shown in Figure 1. The identified metabolites were categorized in the following chemical classes: (i) amino acids (*n* = 7; alanine, glutamate, isoleucine, leucine, phenylalanine, tyrosine, and valine); (ii) saccharides (*n* = 1; glucose); (iii) salts (*n* = 6; acetate, citrate, formate, fumarate, lactate, and malonate); (iv) alcohols (*n* = 2; ethanol and methanol); and (v) other organic compounds (*n* = 5; carnitine, creatine, creatine-phosphate, hypotaurine, myo-inositol, sn-glycero-3-phosphocholine and trimethylamine N-oxide).

**TABLE 1:** Concentration of metabolites (mM) in pig seminal plasma (*n* = 28).

**Metabolite**	**Median**	**Quartile 25**	**Quartile 75**
Acetate	0.1312	0.1142	0.1431
Alanine	0.1257	0.0999	0.1778
Carnitine	0.7736	0.5943	1.1612
Citrate	6.6307	4.6555	8.5421
Creatine	0.3750	0.2487	0.4934
Creatine phosphate	0.0538	0.0433	0.0792
Ethanol	0.2020	0.1701	0.2203
Formate	0.1939	0.1790	0.2192
Fumarate	0.0022	0.0017	0.0032
Glucose	0.1480	0.0824	0.2476
Glutamate	0.4745	0.3590	0.6526
Hypotaurine	1.9639	1.5797	2.5621
Isoleucine	2.7658	2.1357	3.6582
Lactate	0.0477	0.0312	0.0616
Leucine	1.8198	1.3941	2.2803
Malonate	0.0633	0.0439	0.0730
Methanol	0.1106	0.0824	0.1635
Myo-Inositol	0.0557	0.0211	0.1208
Phenylalanine	4.2850	2.8488	5.3341
sn-Glycero-3-phosphocholine	24.3080	19.1166	30.3360
Trimethylamine N-oxide	0.2868	0.1614	0.4411
Tyrosine	0.0237	0.0176	0.0302
Valine	0.1039	0.0795	0.1376

**FIGURE 2:**
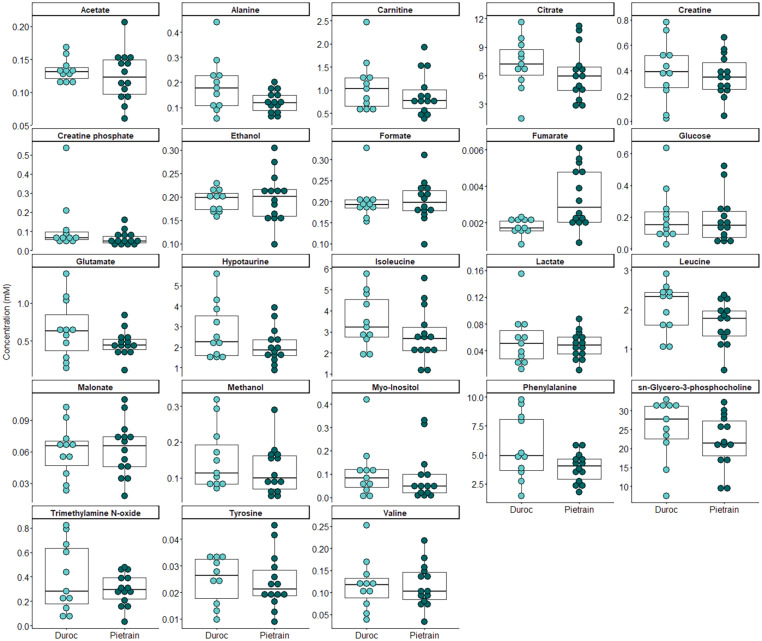
Concentration of metabolites in seminal plasma from ejaculates (one per boar) of different pig breeds (Pietrain, *n* = 14 and Duroc, *n* = 11). Boxes enclose the 25th and 75th percentiles, the whiskers extend to the 5th and 95th percentiles, and the line indicates the median. Each dot represents the metabolite concentration from each ejaculate. Differences between breeds (*P* < 0.05) are represented as ^∗^.

After identifying SP-metabolites, the breed effect on their concentration was evaluated (Supplementary Table 1) for Duroc (*n* = 11) and Pietrain (*n* = 14) breeds. Samples from Tempo and Landrace boars could not be included in the analysis because of the insufficient number of ejaculate samples (for Tempo, *n* = 2, and for Landrace, *n* = 1). Only the concentration of fumarate exhibited significant differences between breeds, the concentration of this metabolite being higher in Pietrain than in Duroc breeds (0.0034 mM vs. 0.0018 mM, *P* < 0.01; Figure 2). These differences in the concentration of fumarate could be assumed to be related to the boar breed due to the fact that all animals were housed in the same building. In fact, in order to evaluate a possible age effect, the Wilcoxon rank sum test was run and evidenced no differences in age distribution between Pietrain and Duroc breeds (*P* > 0.05). Therefore, considering that fumarate could be influenced by the boar breed, this metabolite was excluded from further analyses in order to specifically evaluate the effect of SP-metabolites on sperm physiology.

### Relationship of SP-Metabolites With Sperm Quality and Functionality Parameters Assessed Immediately After Semen Collection

**FIGURE 3:**
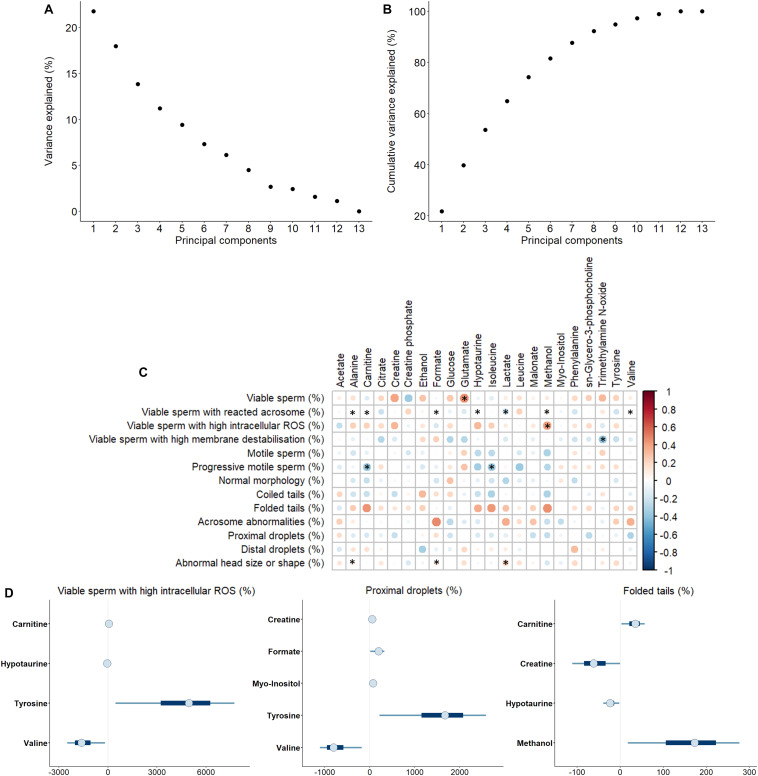
**(A)** Variance explained by each principal component. **(B)** Cumulative variance explained by principal components. **(C)** Correlations between metabolites identified in pig seminal plasma and different sperm functionality parameters analyzed immediately after semen collection. The color saturation of red to blue represents the correlation coefficients (R) between metabolites, from 1 to -1, respectively. Significant correlations (*P* < 0.05) are marked with *. **(D)** Bayesian multiple linear regression models for percentage of viable sperm with high intracellular reactive oxygen species, ROS (H_2_O_2_), percentages of sperm with proximal droplets and percentages of sperm with folded tails. Thin lines represent the 90% credible intervals, and the blue box represents the 50% credible intervals. Dots represent the median estimated for each metabolite in the model.

Sperm quality and functionality was first evaluated immediately after semen collection (Supplementary Table 2). PCA analysis was used to assess if one or few principal components (PC) explained enough variability, so as they could be used to categorize the sample by high and low sperm quality (Figures 3A,B). The results revealed that no PC exceeded the percentage of the variance explained (Figure 3A). Moreover, eight PC were required to achieve a good proportion of the variance explained (>90%; Figure 3B). Because no PC was representative enough for semen quality classification, all sperm parameters were individually analyzed.

Therefore, correlations between the concentration of SP-metabolites and specific sperm quality and functionality parameters were assessed (Figure 3C). Most of the correlations were observed to be from weak (0.00 < R < 0.39) to moderate (0.40 < R < 0.69) (Schober et al., 2018). The statistically significant correlations (*P* < 0.050) with the highest R scores were as follows: i) the percentage of viable sperm was positively correlated with glutamate concentration in SP (R = 0.46, *P* = 0.014); ii) the percentage of viable sperm with high intracellular H_2_O_2_ levels was positively correlated with methanol concentration in SP (R = 0.42, *P* = 0.027); iii) the percentage of viable sperm with high membrane destabilization was negatively correlated with trimethylamine N-oxide concentration in SP (R = −0.38, *P* = 0.045); and iv) the percentage of progressive motile sperm was negatively correlated with both carnitine (R = −0.39, *P* = 0.043) and isoleucine (R = -0.39, *P* = 0.041) concentrations in SP.

After the evaluation of individual correlations between SP-metabolites and sperm quality and functionality parameters, Bayesian multiple linear regression analysis was carried out. This multivariate analysis allows both the development of a potentially predictive model for each parameter and the quantification of the relative contribution of SP-metabolites to the model. Additionally, the use of a Bayesian framework allows the quantification of the uncertainty associated to each coefficient, which provides the whole distribution of the coefficients and not a single point estimate (i.e., the mean). A model was generated for every sperm quality and functionality parameter (Supplementary Figure 1A-M). In general, due to high uncertainties associated to coefficients, relationships between metabolites and sperm quality and functionality indicators could not be reported, except for three variables: i) percentages of viable sperm with high intracellular H_2_O_2_, which were positively influenced by tyrosine and carnitine, and negatively by valine and hypotaurine; ii) percentages of sperm with proximal droplets, which were positively influenced by creatine, formate, myo-inositol and tyrosine, and negatively by valine; and iii) percentages of sperm with folded tails, which were positively influenced by carnitine and methanol, and negatively by creatine and hypotaurine (Figure 3D).

### Relationship Between SP-Metabolites and Sperm Resilience to Liquid Storage at 17°C for 72 h

**FIGURE 4:**
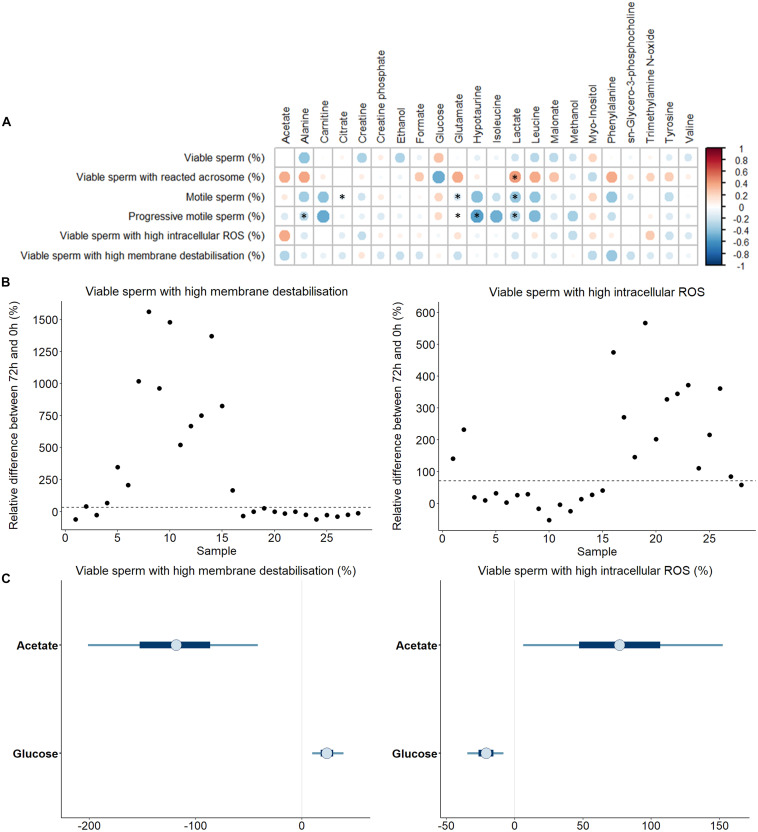
**(A)** Correlations between metabolites identified in pig seminal plasma and different sperm quality and functionality parameters evaluating the quality loss after 72 h of liquid storage at 17 °C. The color saturation of red to blue represents the correlation coefficients (R) between metabolites, from 1 to −1, respectively. Significant correlations (*P* < 0.05) are marked with *. **(B)** Plot showing the relative differences of viable sperm with high membrane destabilization and with high intracellular reactive oxygen species, ROS (H_2_O_2_) for each SP sample. Two different sperm resilience to liquid storage patterns can be observed (marked with a discontinuous line). **(C)** Bayesian logistic regression models for the percentage viable sperm with high membrane destabilization and with high intracellular H_2_O_2_. Thin lines represent the 90% credible intervals, and the blue box represents the 50% credible intervals. Dots represent the median estimated for each metabolite in the model.

Sperm quality and functionality were also assessed after 72 h of liquid storage (Supplementary Table 3). In order to evaluate whether any of the identified SP-metabolites could contribute to explain the sperm ability to withstand liquid storage at 17°C, correlations between concentrations of SP-metabolites and sperm quality and functionality parameters assessed after 72 h at 17°C were calculated (Figure 4A). As observed before, most correlations were found to be weak or moderate. The most relevant and significant (*P* < 0.050) correlations found in terms of higher R scores were: i) the percentage of viable sperm with non-intact acrosome, which was positively correlated with leucine (R = 0.40, *P* = 0.038); ii) the percentage of total motile sperm, which was negatively correlated with leucine (R = −0.40, *P* = 0.039); and iii) the percentage of progressive motile sperm, which was negatively correlated with carnitine (R = −0.41, *P* = 0.031), hypotaurine (R = −0.45, *P* = 0.018), isoleucine (R = −0.47, *P* = 0.014) and leucine (R = −0.43, *P* = 0.024).

Once the correlations between individual sperm parameters and SP-metabolites were analyzed, a Bayesian multiple linear regression analysis was run. Similar to previous findings, most of the models did not show significant contributions of SP-metabolites to sperm quality and functionality parameters after liquid storage at 17°C for 72 h (Supplementary Figures 2A-G). Nevertheless, two parameters, the percentage of viable sperm with high intracellular H_2_O_2_ and the percentage of viable sperm with high plasma membrane destabilization, exhibited two groups with different sperm resilience to liquid storage (Figure 4B). For this reason, logistic regression models were used for these two sperm parameters (Figure 4C). Concentrations of glucose and acetate in SP showed an opposite influence depending on the sperm parameter assessed. Thus, whereas acetate and glucose showed a positive and negative effect on viable sperm with high intracellular H_2_O_2_, respectively, the opposite pattern was observed in viable sperm with high membrane destabilization (i.e., glucose and acetate exhibiting a positive and a negative effect, respectively).

### Correlations Between SP-Metabolites

**FIGURE 5:**
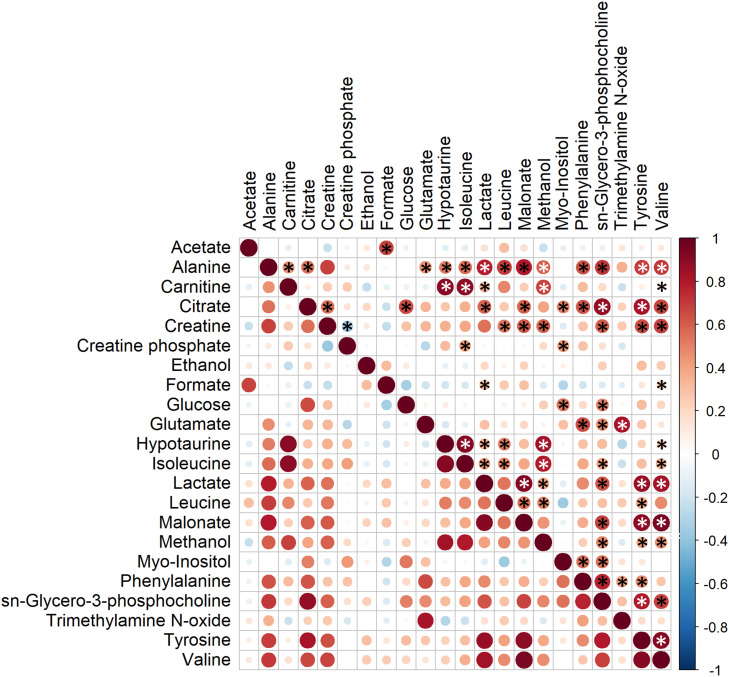
Correlation plot of the twenty-three metabolites identified in boar seminal plasma. The color saturation from red to blue represents the Pearson correlation coefficients (R) between metabolites, from 1 to –1, respectively. Significant correlations (*P* < 0.05) are marked with *.

Correlations between SP-metabolites were also analyzed and observed to vary with different magnitude (Figure 5). Interestingly, all significant correlations between SP-metabolites were found to be positive except for that observed between creatine and creatine phosphate (R = −0.38, *P* < 0.01). The most relevant positive correlations (R > 0.8 and *P* < 0.01; Schober et al., 2018) were: alanine vs. malonate (R = 0.80), carnitine vs. hypotaurine (R = 0.90), carnitine vs. isoleucine (R = 0.89), citrate vs. sn-glycero-3-phosphocholine (R = 0.88), citrate vs. tyrosine (R = 0.82), glutamate vs. trimethylamine N-oxide (R = 0.83), hypotaurine vs. isoleucine (R = 0.92), hypotaurine vs. methanol (R = 0.83), isoleucine vs. methanol (R = 0.80), lactate vs. malonate (R = 0.92), lactate vs. tyrosine (R = 0.86), lactate vs. valine (R = 0.84), sn-glycero-3-phosphocholine vs. tyrosine (R = 0.80), and tyrosine vs. valine (R = 0.91).

## Discussion

As far as we know, this is the first report that performed an in-depth metabolomic profile of pig SP quantifying the identified metabolites. Moreover, this is the first study conducted in a livestock species that explored the putative influence of SP-metabolites on sperm physiology and on the sperm resilience to liquid storage at 17 °C. Specifically, this exploratory study reported that: i) a total of 23 SP-metabolites were present at measurable concentrations in pig SP; ii) except for fumarate, no differences in SP-metabolites concentration were identified between pig breeds (Duroc and Pietrain); iii) glutamate, methanol, trimethylamine N-oxide, carnitine and isoleucine concentrations in SP were related to specific sperm quality and functionality parameters evaluated right after semen collection; iv) leucine, hypotaurine, carnitine and isoleucine concentrations in SP were found to be related with the sperm ability to withstand liquid storage; and v) Bayesian multiple regression models allowed the identification of putative metabolite patterns for specific sperm parameters. In addition, very strong relationships between SP-metabolites were also observed.

Metabolomic approaches allow the study of cellular metabolic products; these low-molecular-weight molecules can ultimately unveil the phenotype of the studied system (Deepinder et al., 2007). For this reason, studying the metabolome has been widely proposed as a promising tool for spermatology and male infertility diagnosis (Aitken, 2010; Kovac et al., 2013; Minai-Tehrani et al., 2016; Bracewell-Milnes et al., 2017; Moura et al., 2018). The NMR spectroscopy is one of the most used technologies in this field, because it provides a non-biased uniform detection of equal sensibility for all proton-containing molecules in samples, and requires little or no processing (Goodacre et al., 2004; Emwas et al., 2019). This analytical method has been widely used for SP-metabolite profiling of several mammalian species, including humans (Hamamah et al., 1993; Segalen et al., 1995; Gupta et al., 2011; Jayaraman et al., 2014; Bonechi et al., 2015; Mumcu et al., 2020), cattle (Kumar et al., 2015) and pigs (Kalic et al., 1997; Mateo-Otero et al., 2020). The present work identified and quantified a total of 23 metabolites in pig SP, including amino acids, saccharides, salts, alcohols, and other compounds. Compared to our previous work (Mateo-Otero et al., 2020), the present study quantified SP-metabolites, as well as identified new molecules (i.e., formate, fumarate, hypotaurine, isoleucine, malonate, myo-inositol, and trimethylamine N-oxide). These new findings may result from improvements in sample handling and purification, and the higher frequency and resolution of the NMR spectrometer. Additionally, while SP in pigs showed a metabolite profile similar to that reported in humans (Hamamah et al., 1993; Segalen et al., 1995; Gupta et al., 2011; Jayaraman et al., 2014; Bonechi et al., 2015; Mumcu et al., 2020), it differed from that found in cattle (Kumar et al., 2015). This was already reported in our previous work (Mateo-Otero et al., 2020) and was confirmed in the present study.

Regarding the new SP-metabolites identified in the present work, both isoleucine and malonate were also identified in the human SP (Gupta et al., 2011; Bonechi et al., 2015; Mumcu et al., 2020). Moreover, only some of the NMR studies carried out in humans quantified the identified metabolites (Gupta et al., 2011, 2013; Bonechi et al., 2015), generally reporting higher SP-metabolite concentrations than those found herein for pigs (Hamamah et al., 1993; Segalen et al., 1995; Gupta et al., 2011; Jayaraman et al., 2014; Bonechi et al., 2015; Mumcu et al., 2020). Variations in SP metabolite profile or in their concentrations could contribute to reveal physiological differences between species in terms of sperm functionality and fertility. Nevertheless, sample handling and processing could also serve to explain these qualitative and quantitative discrepancies.

As aforementioned, one of the most relevant results of the present study was that concentrations of 23 metabolites present in pig SP were uncovered. In addition, we observed that concentrations of SP-metabolites varied between ejaculates. Since all boars had similar age, were housed under the same conditions, and were fed with the same commercial diet, we hypothesize that such variability could be related to pig breeds. However, when samples were divided considering breeds, no effect on SP-metabolites concentration was observed, except for fumarate, which exhibited higher levels in Pietrain than in Duroc boars. This agrees with most of the studies evaluating SP components in samples from different pig breeds, in which no breed effect was reported (Barranco et al., 2015b, 2016, 2020; Llavanera et al., 2020). However, since the sample size for each breed was limited in the present study, further research should verify this assumption. Nevertheless, considering our findings, one could assume that differences in the concentration of SP-metabolites found between boars could contribute to explain the disparity observed in their sperm quality parameters assessed immediately after semen collection or in their sperm resilience to liquid storage.

Many efforts have been focused on exploring pig SP composition with the aim to uncover SP biomarkers able to predict semen quality after ejaculate collection (López Rodríguez et al., 2012; Barranco et al., 2016, 2017, 2020; Llavanera et al., 2020). In the last years, the advances in metabolomic analytical methods have allowed the identification of SP-metabolites as potential sperm quality biomarkers in mammalian species, such as humans (Bonechi et al., 2015; Engel et al., 2019; Wang et al., 2019; Mumcu et al., 2020; Xu et al., 2020) and cattle (Moura et al., 2018). Our results suggest that metabolomic analysis of pig SP could offer a new source of sperm functionality biomarkers. Therefore, by using an exploratory approach, the present study evaluated the potential relationship of the concentration of SP-metabolites with sperm quality and functionality parameters assessed immediately after semen collection. While a strongly significant (*P* = 0.014) positive correlation between the concentration of glutamate in SP and sperm viability was observed, this does not fully agree with previous findings in which the addition of monosodium glutamate to fresh pig semen had no effect on the percentage of viable sperm during *in vitro* capacitation (Spinaci et al., 2017). On the other hand, a positive relationship (*P* = 0.027) between methanol concentration in SP and viable sperm with high H_2_O_2_ was found. These results do not come as a surprise when one considers the well-known oxidative stress-inducer role of this metabolite (Parthasarathy et al., 2006). In addition, a negative relationship (*P* = 0.045) between trimethylamine N-oxide concentration in SP and viable sperm with high membrane destabilization was detected. These results would be in agreement with the study by Darbandi et al. (2019) in men, who found that higher levels of this metabolite in human SP were related with high intracellular ROS levels in sperm and could ultimately cause membrane lipid peroxidation and, thus, membrane destabilization. Finally, our data indicated that concentrations of both carnitine (*P* = 0.043) and isoleucine (*P* = 0.041) in SP were negatively correlated to progressively motile sperm. Regarding carnitine, our results are in agreement with previous studies in humans using liquid chromatography-mass spectrometry (LC-MS) (Wang et al., 2019; Xu et al., 2020). This metabolite was found to be a potential SP-biomarker of poor semen quality (Wang et al., 2019), and to be positively related with sperm deformity rate (Xu et al., 2020). By contrast, our findings do not agree with the previous literature, since several studies reported the beneficial effect of supplementing diet with L-carnitine on sperm progressive motility in patients with different types of infertility (Lenzi et al., 2004; Garolla et al., 2005; Micic et al., 2019). On the other hand, a negative effect of isoleucine on sperm has been observed in buffalos, since poor semen quality is associated to low levels of isoleucine (Binsila et al., 2020). Our data indicated that each relationship has a different *P* value; while glutamate and sperm viability were found to have a very robust correlation coefficient (*P* = 0.014), the others ranged between *P* = 0.020 and *P* = 0.045. In this latter case, and despite the fact that these correlations had a high degree of confidence (> 95%), one could not exclude that some happened by chance (given that type I error increases with the number of correlations tested). For this reason, except for glutamate, the other relationships should be considered as a preliminary approximation to elucidate the effect of these SP-metabolites on sperm physiology. Thus, further experiments to validate the correlations observed in the present study are warranted. What is more, considering the aforementioned discrepancies between species, the actual effect of these metabolites on pig sperm needs to be addressed in future studies because species-specific regulation of SP-metabolites could be observed. Another possibility to explain such discrepancies could be that only moderate correlations were found between pig SP-metabolites and sperm quality parameters. Hence, specificity and sensibility of SP-metabolites should be tested and validated before their use as biomarkers.

This study was also able to explore whether concentrations of metabolites in SP were able to explain variations in sperm quality/functionality parameters using Bayesian multiple linear regression models. Only metabolites whose 90% credible intervals did not cross 0 in a Pareto distribution were considered as valid and, thus, as promising biomarkers. We did not find clear patterns for most of the quality parameters evaluated immediately after semen collection. However, models based on viable sperm with high intracellular ROS, sperm with proximal droplets and sperm with folded tails showed a positive or negative effect of specific SP-metabolites. Most relationships between metabolites have not yet been described in the literature; thus, further studies focused on determining whether these relationships have a biological significance are required.

Sperm quality of pig seminal AI-doses gradually declines during their storage at 17°C (Waberski et al., 2011; Yeste, 2017), showing differences in their sperm resilience to liquid preservation (Parrilla et al., 2012; Zakošek Pipan et al., 2014; Roca et al., 2015). While several studies have demonstrated that some SP-proteins are able to predict pig sperm resilience to liquid storage (Zakošek Pipan et al., 2014; Barranco et al., 2016, 2019; Parrilla et al., 2019; Llavanera et al., 2020), no study has, to the best of our knowledge, evaluated the potential relationship between SP-metabolite composition and the sperm ability to sustain 72 h of liquid storage. Our approach suggested that some SP-metabolites were positively and negatively related with the sperm functionality decline during liquid preservation. Leucine concentration in SP was found to be positively correlated with the percentage of viable sperm with a non-intact acrosome (*P* = 0.038) and negatively associated with the loss of total (*P* = 0.039) and progressive (*P* = 0.024) motility after 72 h of liquid storage. Despite the fact that no information regarding leucine effect on sperm performance is available in mammals, the addition of this metabolite is known to have a positive effect on the motility of zebrafish sperm (Zhang et al., 2017). Similarly, our findings revealed that hypotaurine concentration in SP was negatively related with higher loss of progressive motility (*P* = 0.018) after liquid storage at 17 °C. In this context, it is worth mentioning that hypotaurine supplementation has been reported to exert a positive impact upon sperm motility in mammalian species, such as hamsters (Boatman et al., 1990) or humans (Holmes et al., 1992; Brugnon et al., 2013). Finally, both carnitine (*P* = 0.031) and isoleucine (*P* = 0.014) were found to be negatively related with the loss of progressive motility after liquid storage. While information regarding the effect of isoleucine on sperm motility is yet to be reported, a study on L-carnitine supplementation during liquid storage of pig semen demonstrated that this metabolite could maintain sperm motility (Yang et al., 2020). Whilst all these data suggest that these metabolites may be promising biomarkers for motility prediction of seminal AI-doses after liquid storage, the different *P* values may, as pointed out before, imply different robustness in the relationships observed. In this regard, while correlations of isoleucine and hypotaurine with loss of progressive motility over liquid storage were high, those with *P* values oscillating between 0.020 and 0.040 would need further validation. For this reason, although our study can be considered as a first steppingstone in establishing biomarkers for the sperm resilience to liquid storage, further experiments are needed to confirm the exact role of SP-metabolites during that process.

In a similar fashion to the case of sperm quality evaluated immediately after semen collection, Bayesian multiple linear regression models did not identify clear metabolite concentration patterns explaining sperm quality loss. However, the percentage of viable sperm with high intracellular H_2_O_2_ and the percentage of viable sperm with high plasma membrane destabilization showed two distinct groups of low- and high-quality loss. For this reason, logistic regression models were considered to be appropriate to further study such relationships. Interestingly, concentrations of acetate and glucose in SP were observed to have opposite effects on both parameters. These findings are in agreement with the literature, as acetate production from glucose through pyruvate has been previously reported to occur in mammalian mitochondria (Liu et al., 2018). Our models showed that low levels of glucose and high levels of acetate have an impact on the percentage of viable sperm with high levels of ROS, which could be explained by the link between acetate production and ROS generation (Liu et al., 2018). On the other hand, glucose could also be involved in fatty acids and cholesterol synthesis (Wolfe, 1998). As we found that high levels of glucose and low levels of acetate influenced the percentage of viable sperm with high membrane destabilization, further studies should explore the exact mechanism that would underlie such observation.

In conclusion, the use of NMR spectroscopy analysis allowed the identification and quantification of 23 metabolites in pig SP. Our exploratory approach was able to suggest that the concentration of selected SP-metabolites was related to sperm quality and functionality parameters of liquid-stored semen samples and to the sperm ability to withstand liquid storage, which led to uncover potential biomarkers. Specifically, the concentration of glutamate in SP appeared to be related to sperm viability when evaluated immediately after ejaculate collection. Moreover, while concentrations of hypotaurine and isoleucine in SP could be involved in the sperm resilience to liquid storage, the pattern observed for glucose and acetate suggests that they could also play a role. Yet, these relationships can only be considered as a first steppingstone for metabolite biomarker identification, as they are pending to be validated in future studies. Nevertheless, this could have a very relevant impact on AI industry because the identification of such metabolites could contribute to improve: i) quality assessment of seminal AI-doses prior to AI; and ii) AI extenders through supplementation with specific metabolites to improve the sperm resilience to liquid storage. Moreover, further research including more semen samples should be conducted to determine whether these SP-metabolites could also be used as *in vivo* fertility biomarkers.

## Data Availability Statement

The raw data supporting the conclusions of this article will be made available by the authors, without undue reservation.

## Ethics Statement

Ethical review and approval for animal participants was not required as samples were provided by the AI-Centre and authors did not manipulate any animals.

## Author Contributions

YM-O, MY, and IB: conceptualization. YM-O, PF-L, and IB: methodology. YM-O, PF-L, JR-M, MY, and IB: formal analysis and investigation. YM-O: writing – original draft preparation. JR-M, JM, JR, MY, and IB: writing – review and editing. JM, JR, and MY: funding acquisition. IB and MY: supervision. All authors have read and agreed to the published version of the manuscript.

## Conflict of Interest

The authors declare that the research was conducted in the absence of any commercial or financial relationships that could be construed as a potential conflict of interest.

## Footnotes

1 https://www.r-project.org/

## Supplementary Material

Supplementary Figure 1 **(A-M)**. Complete Bayesian multiple linear regression models for all the parameters evaluated immediately after semen collection.

Supplementary Figure 2 **(A-F)** Complete Bayesian multiple linear regression models for all the parameters evaluated immediately after semen collection. **(G,H)** Complete Bayesian logistic regression models for percentages of viable sperm with high membrane destabilization, and with high intracellular Ros.

Supplementary Table 1 Metabolite concentrations (mM) in seminal plasma of Pietrain and Duroc breeds. Significant correlations (*P < 0.05*) are marked with ^∗^.

Supplementary Table 2 Sperm quality and functionality parameters of pig semen samples (*n = 28*) assessed immediately after semen collection. Sd: Standard Deviation.

Supplementary Table 3 Sperm quality loss of pig semen samples (*n = 28*) after 72 h of liquid storage at 17°C. Sd: Standard Deviation.
