# Supplementary material for: Metabolite Profiling of Pig Seminal Plasma Identifies Potential Biomarkers for Sperm Resilience to Liquid Preservation
Source: Front Cell Dev Biol. 2021 May 28;9:669974. doi: 10.3389/fcell.2021.669974 (PMC8194698; doi:10.3389/fcell.2021.669974)
Supplement: Supplementary Figure 1 — (A-M). Complete Bayesian multiple linear regression models for all the parameters evaluated immediately after semen collection. [file Presentation_1.pdf]

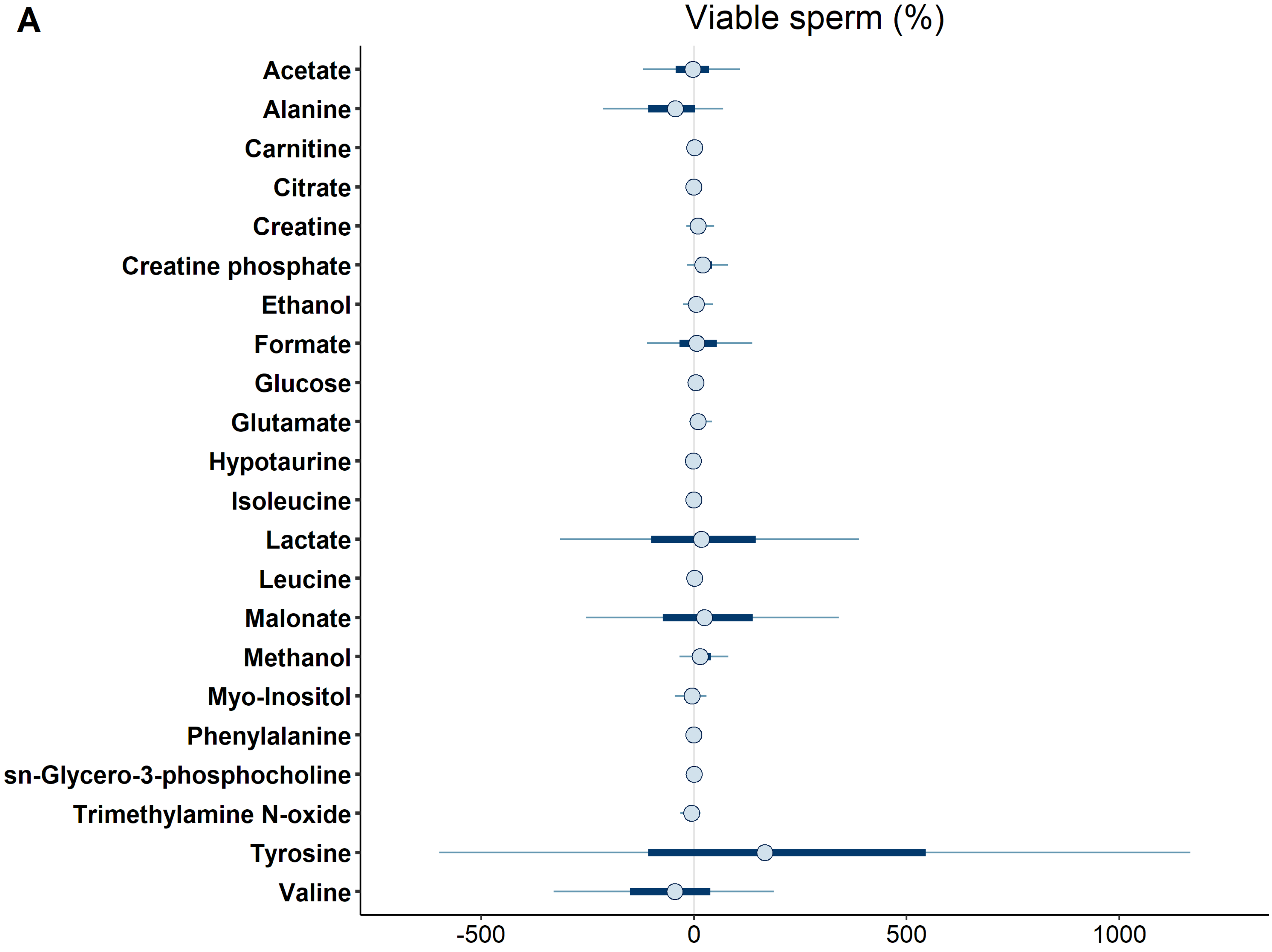

A
Viable sperm (%)
Acetate
Alanine
Carnitine
Citrate
Creatine
Creatine phosphate
Ethanol
Formate
Glucose
Glutamate
Hypotaurine
Isoleucine
Lactate
Leucine
Malonate
Methanol
Myo-Inositol
Phenylalanine
sn-Glycero-3-phosphocholine
Trimethylamine N-oxide
Tyrosine
Valine
-500
0
500
1000

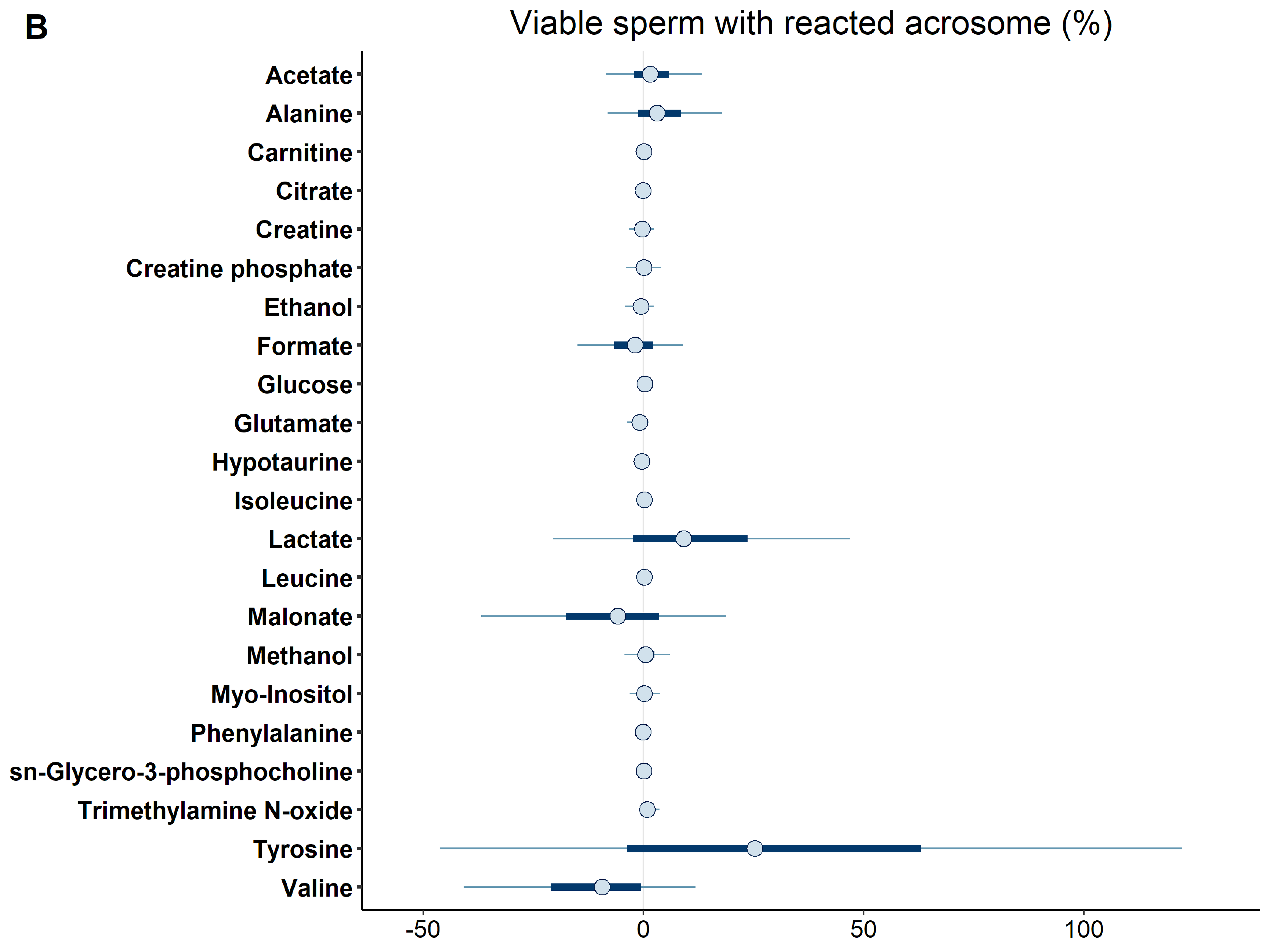
B
Viable sperm with reacted acrosome (%)
Acetate
Alanine
Carnitine
Citrate
Creatine
Creatine phosphate
Ethanol
Formate
Glucose
Glutamate
Hypotaurine
Isoleucine
Lactate
Leucine
Malonate
Methanol
Myo-Inositol
Phenylalanine
sn-Glycero-3-phosphocholine
Trimethylamine N-oxide
Tyrosine
Valine
-50
0
50
100

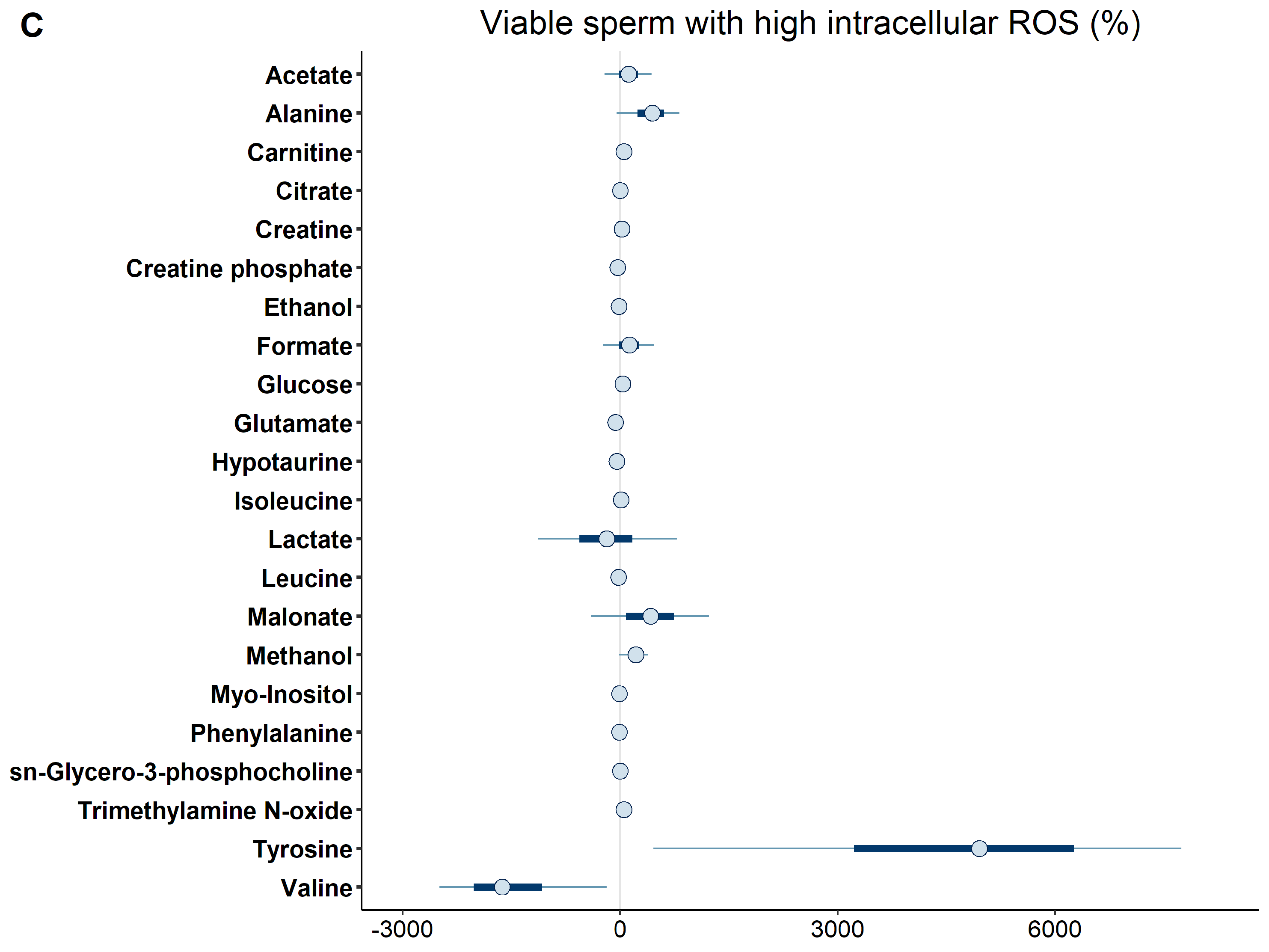
C
Viable sperm with high intracellular ROS (%)
Acetate
Alanine
Carnitine
Citrate
Creatine
Creatine phosphate
Ethanol
Formate
Glucose
Glutamate
Hypotaurine
Isoleucine
Lactate
Leucine
Malonate
Methanol
Myo-Inositol
Phenylalanine
sn-Glycero-3-phosphocholine
Trimethylamine N-oxide
Tyrosine
Valine
-3000
0
3000
6000

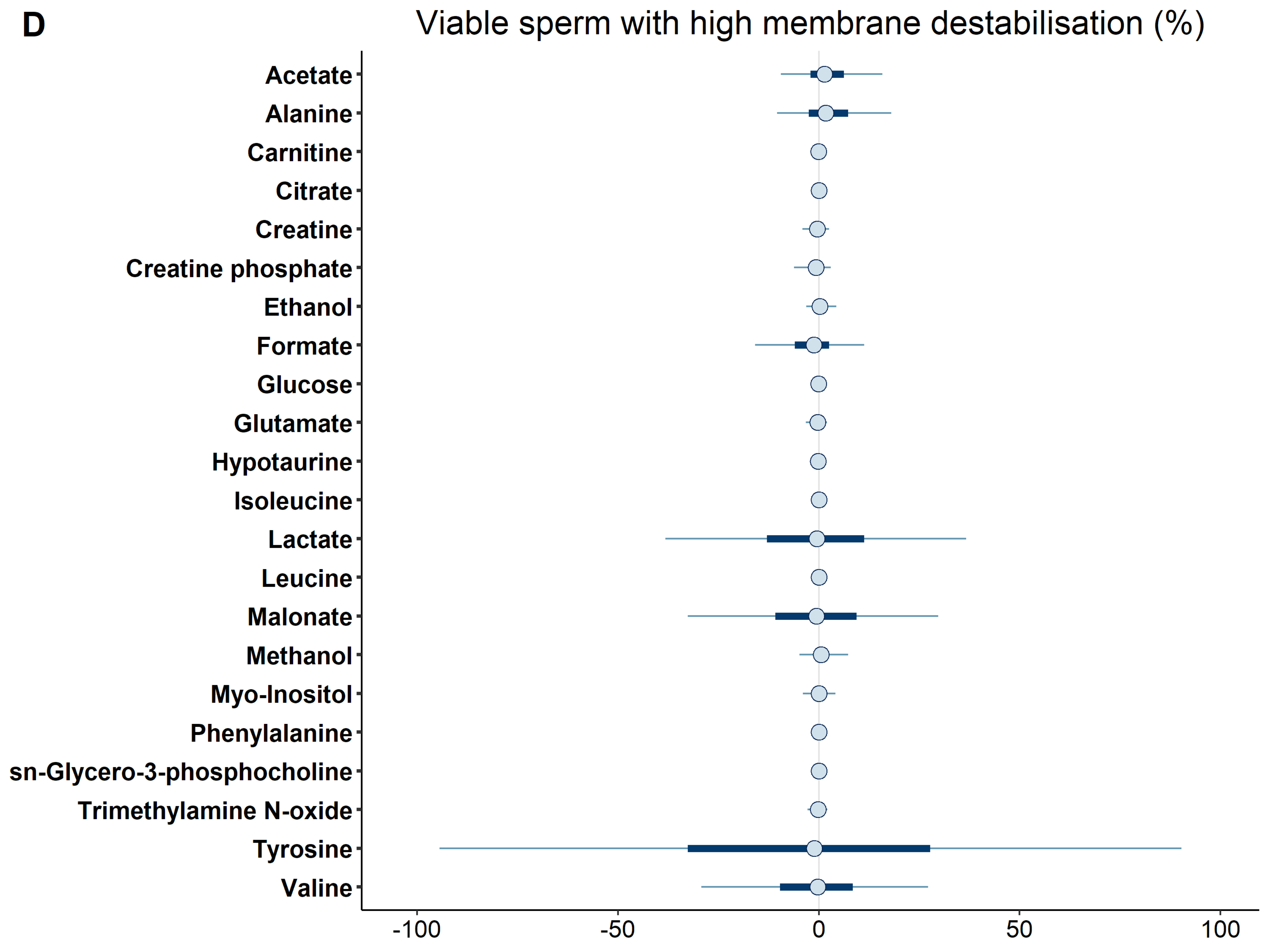
D
Viable sperm with high membrane destabilisation (%)
Acetate
Alanine
Carnitine
Citrate
Creatine
Creatine phosphate
Ethanol
Formate
Glucose
Glutamate
Hypotaurine
Isoleucine
Lactate
Leucine
Malonate
Methanol
Myo-Inositol
Phenylalanine
sn-Glycero-3-phosphocholine
Trimethylamine N-oxide
Tyrosine
Valine
-100
-50
0
50
100

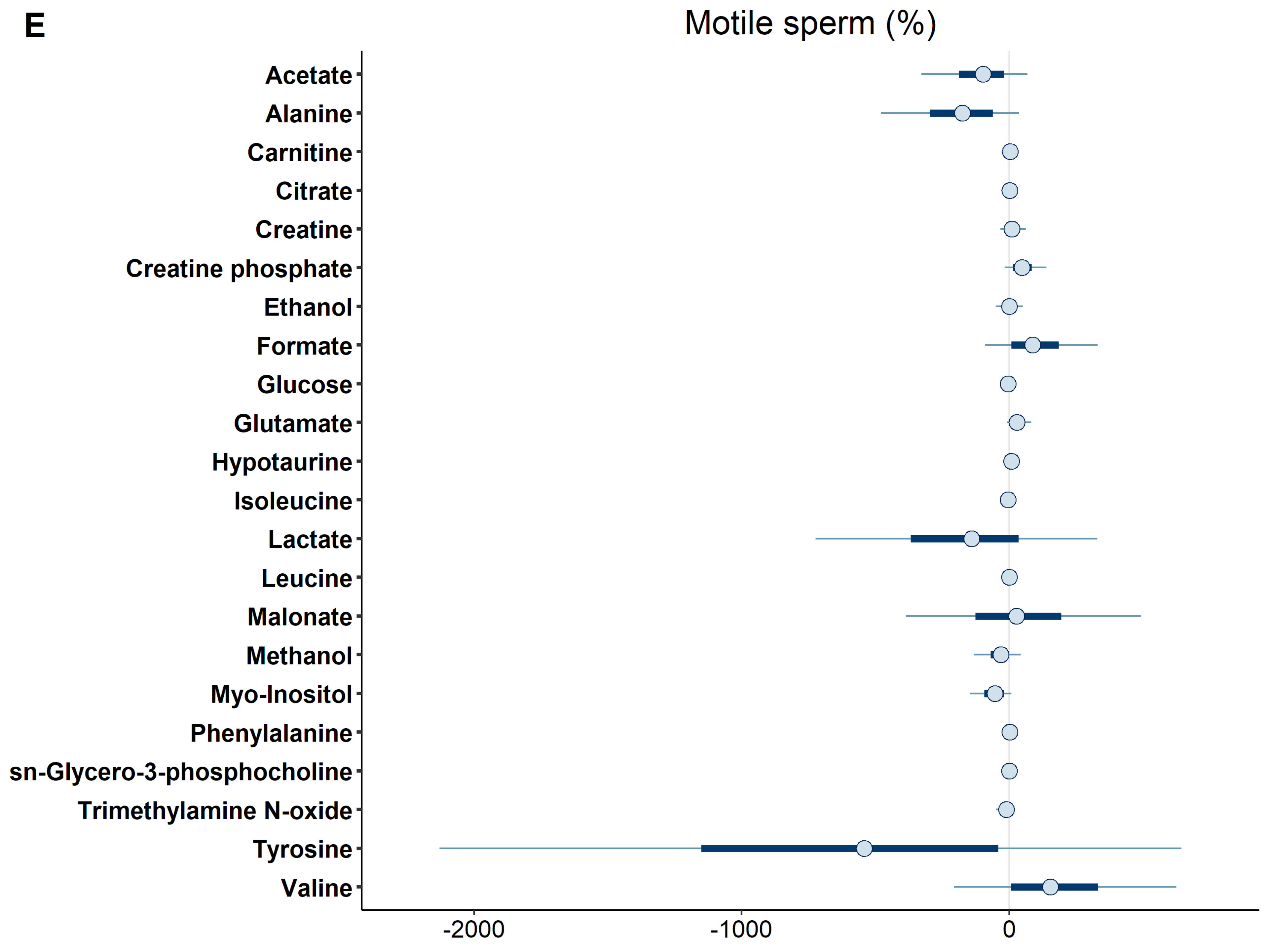
E
Motile sperm (%)
Acetate
Alanine
Carnitine
Citrate
Creatine
Creatine phosphate
Ethanol
Formate
Glucose
Glutamate
Hypotaurine
Isoleucine
Lactate
Leucine
Malonate
Methanol
Myo-Inositol
Phenylalanine
sn-Glycero-3-phosphocholine
Trimethylamine N-oxide
Tyrosine
Valine
-2000
-1000
0

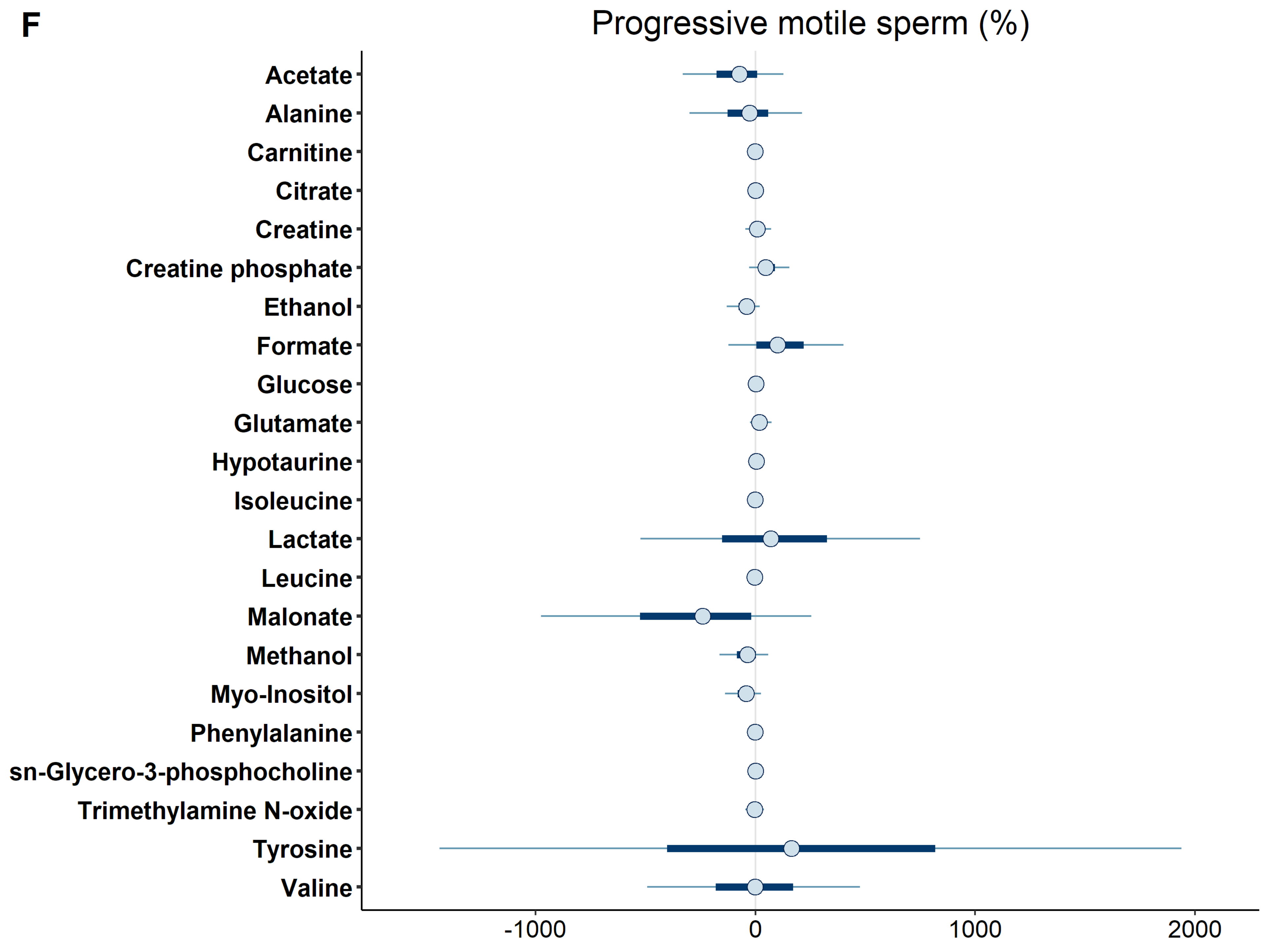
F
Progressive motile sperm (%)
Acetate
Alanine
Carnitine
Citrate
Creatine
Creatine phosphate
Ethanol
Formate
Glucose
Glutamate
Hypotaurine
Isoleucine
Lactate
Leucine
Malonate
Methanol
Myo-Inositol
Phenylalanine
sn-Glycero-3-phosphocholine
Trimethylamine N-oxide
Tyrosine
Valine
-1000
0
1000
2000

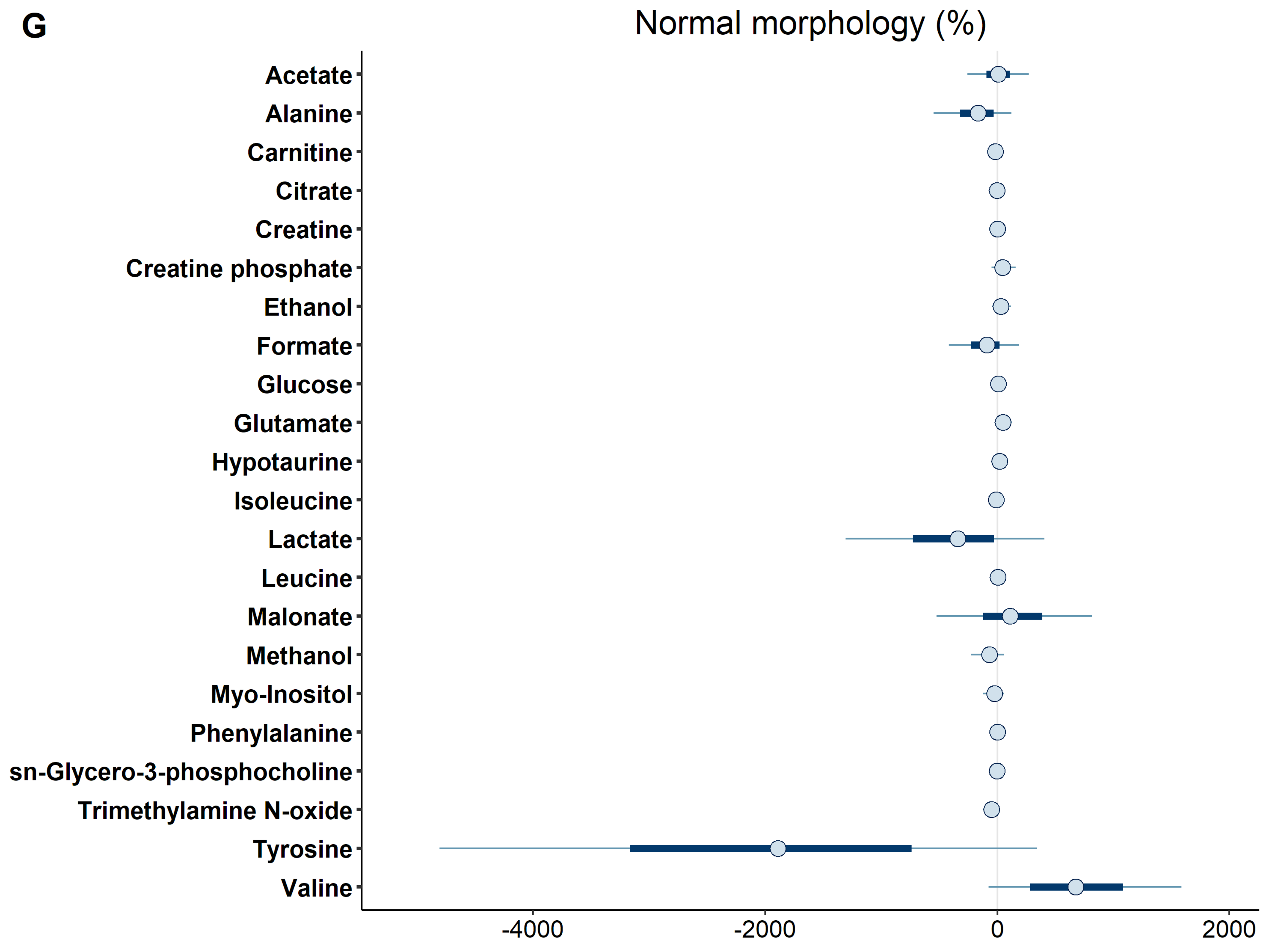

G
Normal morphology (%)
Acetate
Alanine
Carnitine
Citrate
Creatine
Creatine phosphate
Ethanol
Formate
Glucose
Glutamate
Hypotaurine
Isoleucine
Lactate
Leucine
Malonate
Methanol
Myo-Inositol
Phenylalanine
sn-Glycero-3-phosphocholine
Trimethylamine N-oxide
Tyrosine
Valine
-4000
-2000
0
2000

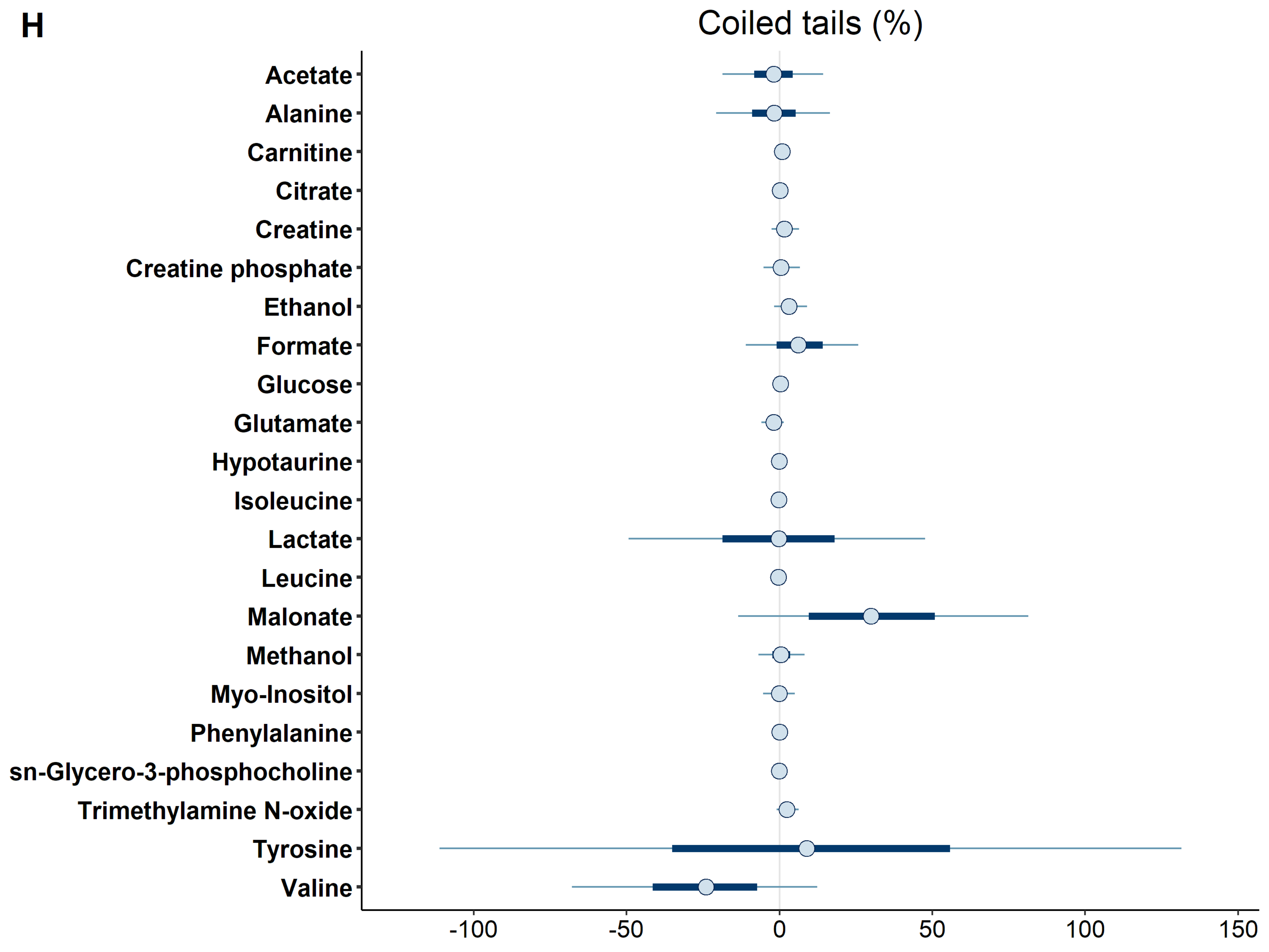
H
Coiled tails (%)
Acetate
Alanine
Carnitine
Citrate
Creatine
Creatine phosphate
Ethanol
Formate
Glucose
Glutamate
Hypotaurine
Isoleucine
Lactate
Leucine
Malonate
Methanol
Myo-Inositol
Phenylalanine
sn-Glycero-3-phosphocholine
Trimethylamine N-oxide
Tyrosine
Valine
-100
-50
0
50
100
150

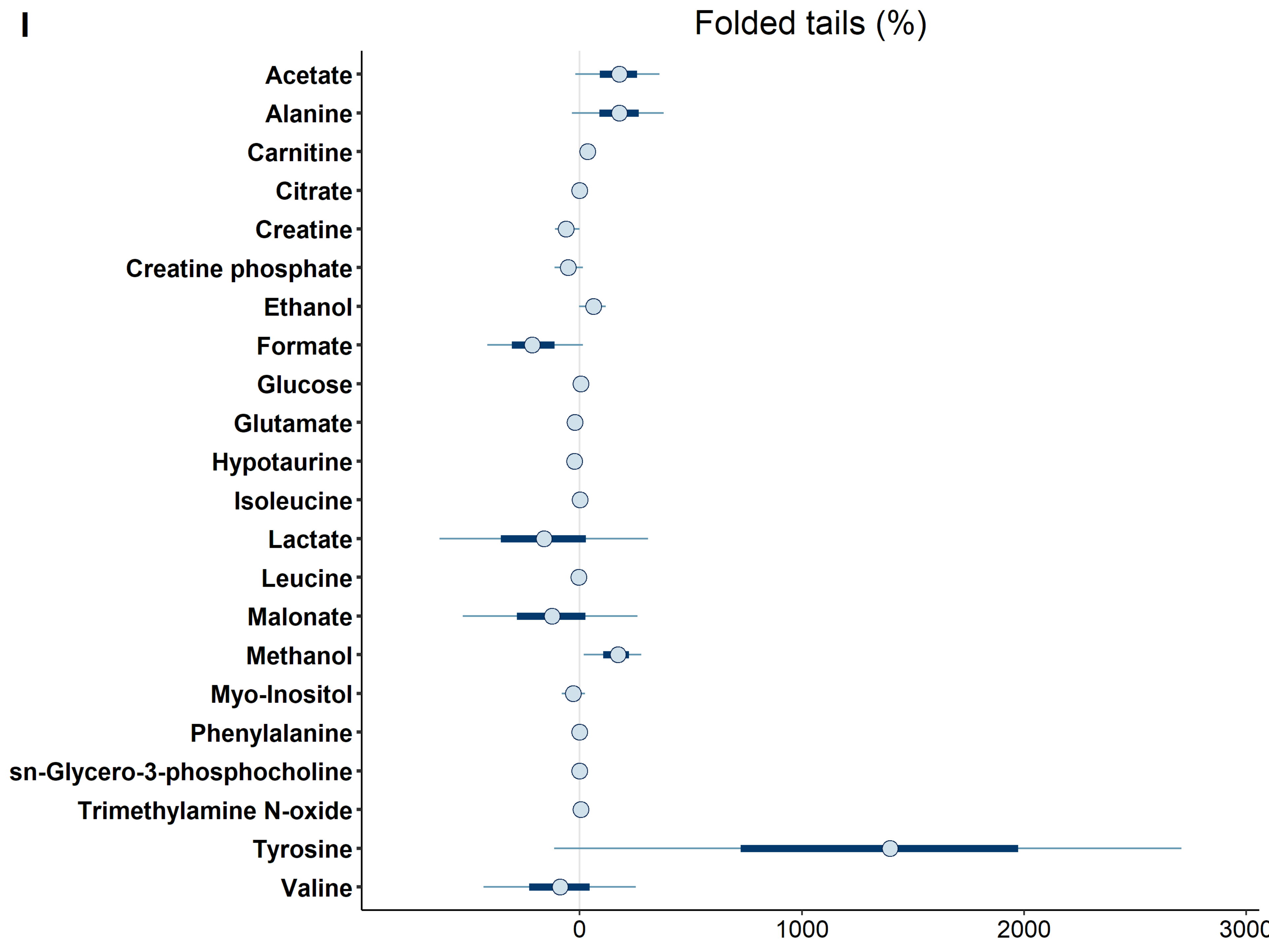
I
Folded tails (%)
Acetate
Alanine
Carnitine
Citrate
Creatine
Creatine phosphate
Ethanol
Formate
Glucose
Glutamate
Hypotaurine
Isoleucine
Lactate
Leucine
Malonate
Methanol
Myo-Inositol
Phenylalanine
sn-Glycero-3-phosphocholine
Trimethylamine N-oxide
Tyrosine
Valine
0
1000
2000
300

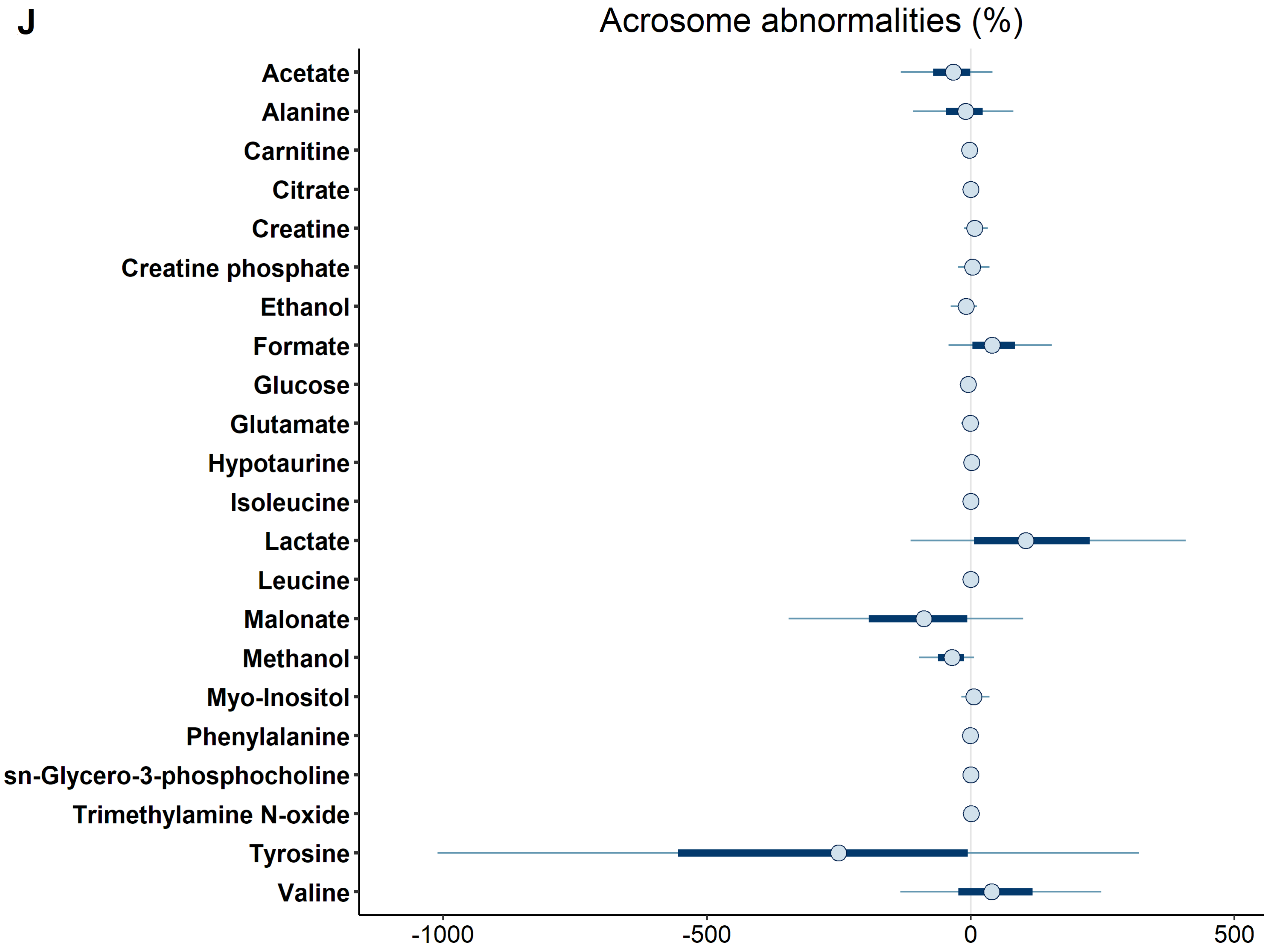
J
Acrosome abnormalities (%)
Acetate
Alanine
Carnitine
Citrate
Creatine
Creatine phosphate
Ethanol
Formate
Glucose
Glutamate
Hypotaurine
Isoleucine
Lactate
Leucine
Malonate
Methanol
Myo-Inositol
Phenylalanine
sn-Glycero-3-phosphocholine
Trimethylamine N-oxide
Tyrosine
Valine
-1000
-500
0
500

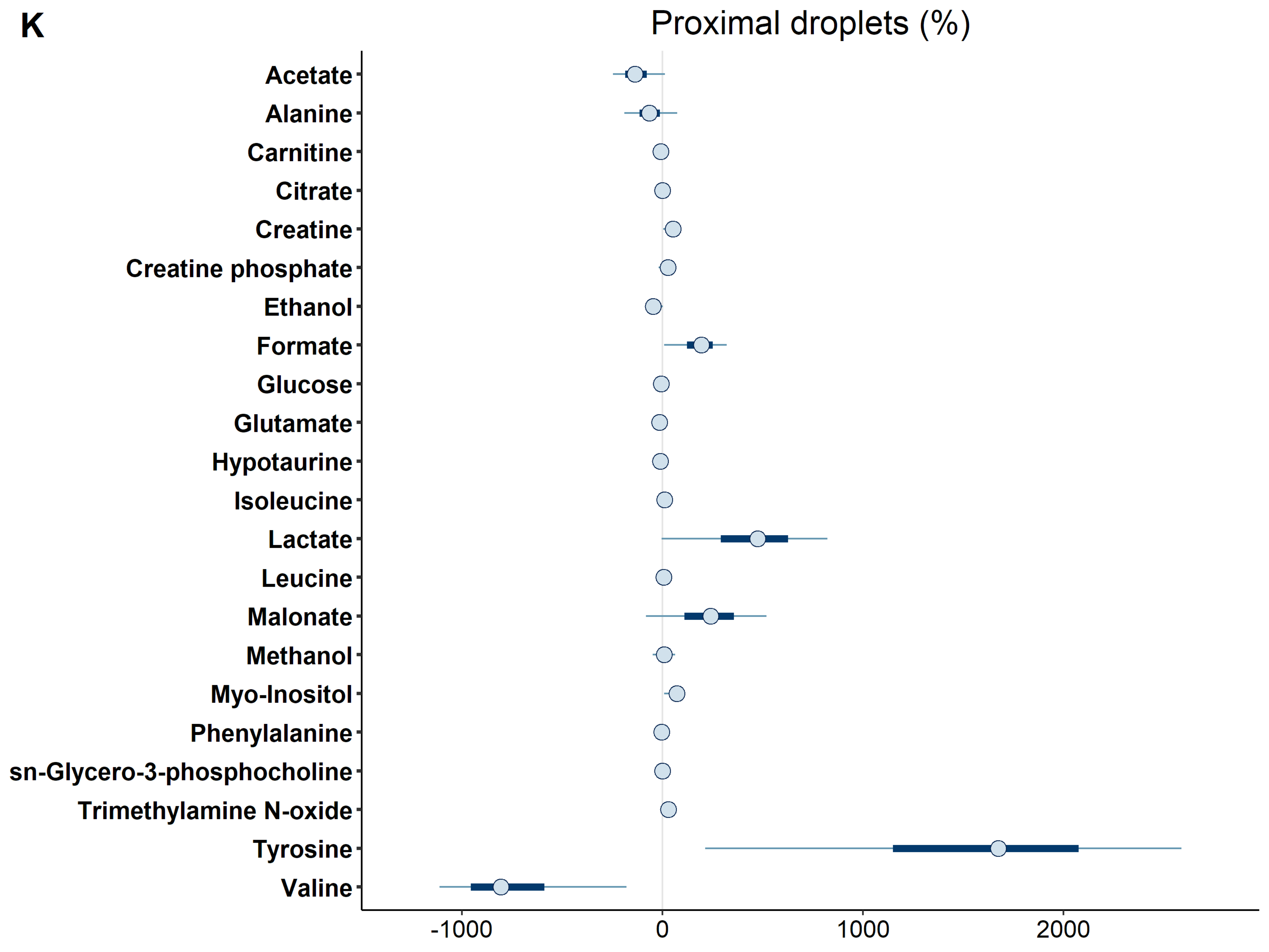

K
Proximal droplets (%)
Acetate
Alanine
Carnitine
Citrate
Creatine
Creatine phosphate
Ethanol
Formate
Glucose
Glutamate
Hypotaurine
Isoleucine
Lactate
Leucine
Malonate
Methanol
Myo-Inositol
Phenylalanine
sn-Glycero-3-phosphocholine
Trimethylamine N-oxide
Tyrosine
Valine
-1000
0
1000
2000

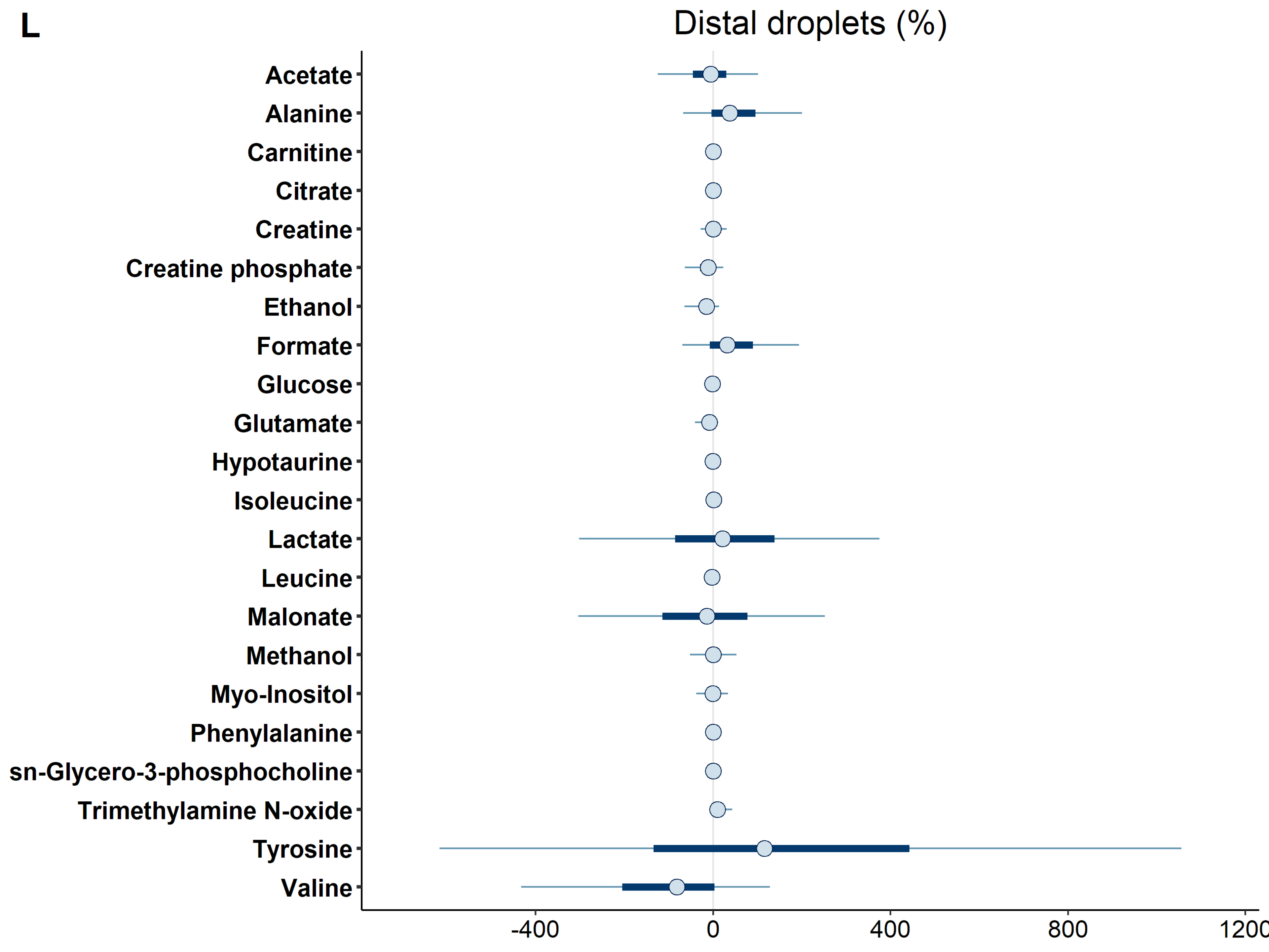

L
Distal droplets (%)
Acetate
Alanine
Carnitine
Citrate
Creatine
Creatine phosphate
Ethanol
Formate
Glucose
Glutamate
Hypotaurine
Isoleucine
Lactate
Leucine
Malonate
Methanol
Myo-Inositol
Phenylalanine
sn-Glycero-3-phosphocholine
Trimethylamine N-oxide
Tyrosine
Valine
-400
0
400
800
1200

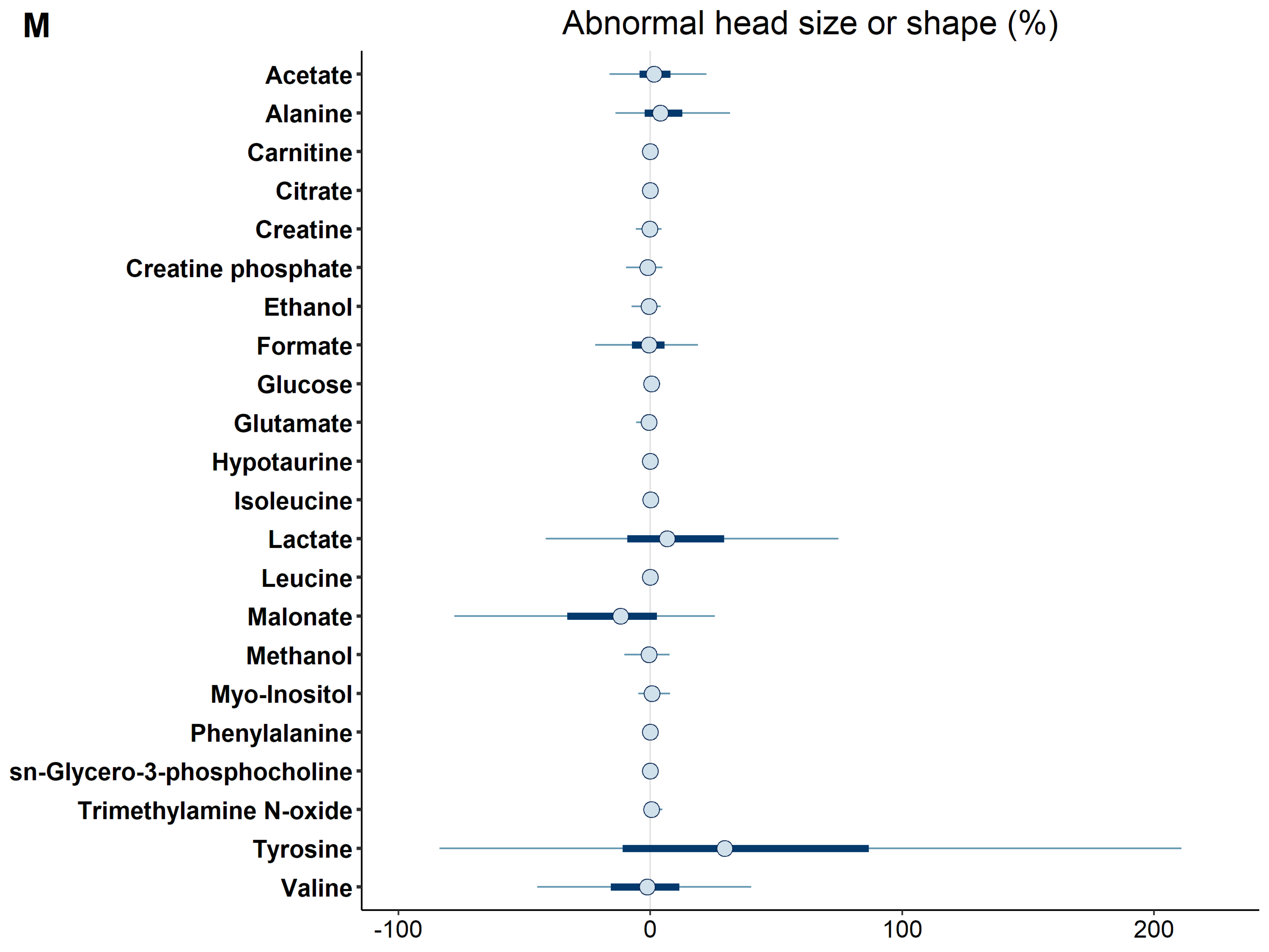
M
Abnormal head size or shape (%)
Acetate
Alanine
Carnitine
Citrate
Creatine
Creatine phosphate
Ethanol
Formate
Glucose
Glutamate
Hypotaurine
Isoleucine
Lactate
Leucine
Malonate
Methanol
Myo-Inositol
Phenylalanine
sn-Glycero-3-phosphocholine
Trimethylamine N-oxide
Tyrosine
Valine
-100
0
100
200
