# Supplementary material for: Metabolite Profiling of Pig Seminal Plasma Identifies Potential Biomarkers for Sperm Resilience to Liquid Preservation
Source: Front Cell Dev Biol. 2021 May 28;9:669974. doi: 10.3389/fcell.2021.669974 (PMC8194698; doi:10.3389/fcell.2021.669974)
Supplement: Supplementary Figure 2 — (A-F) Complete Bayesian multiple linear regression models for all the parameters evaluated immediately after semen collection. (G,H) Complete Bayesian logistic regression models for percentages of viable sperm with high membrane destabilization, and with high intracellular Ros. [file Presentation_2.pdf]

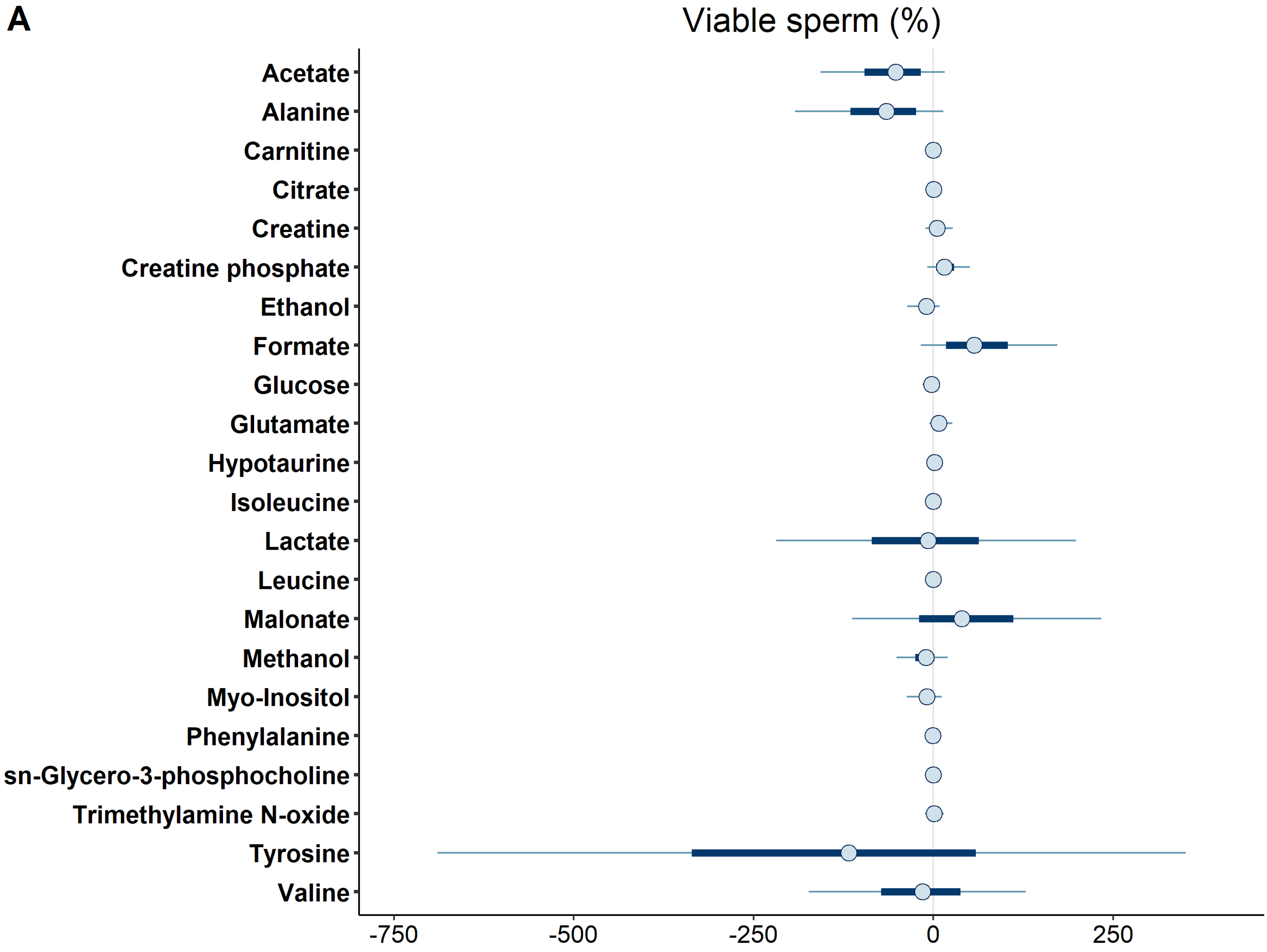

A
Viable sperm (%)
Acetate
Alanine
Carnitine
Citrate
Creatine
Creatine phosphate
Ethanol
Formate
Glucose
Glutamate
Hypotaurine
Isoleucine
Lactate
Leucine
Malonate
Methanol
Myo-Inositol
Phenylalanine
sn-Glycero-3-phosphocholine
Trimethylamine N-oxide
Tyrosine
Valine
-750
-500
-250
0
250

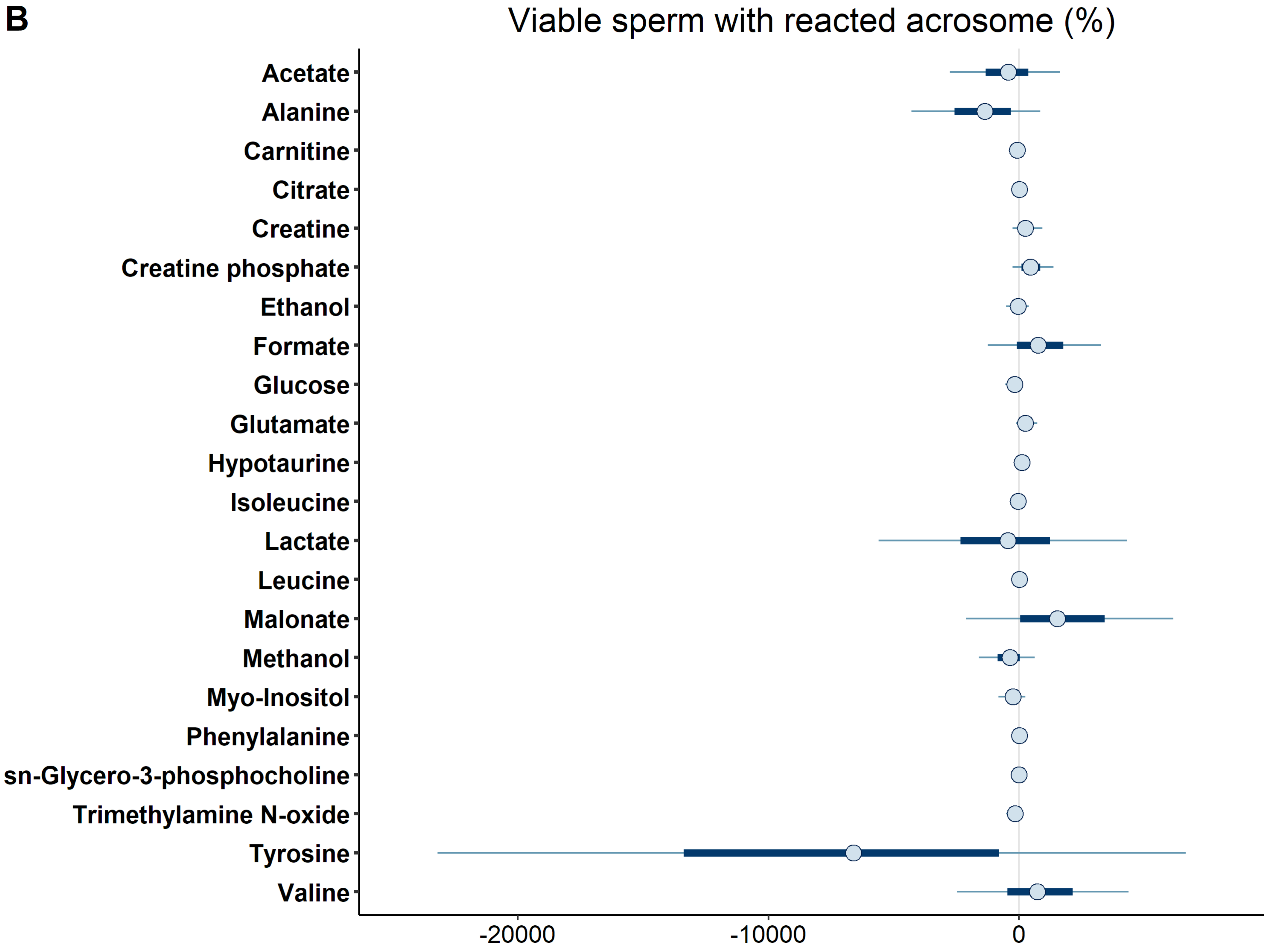
B
Viable sperm with reacted acrosome (%)
Acetate
Alanine
Carnitine
Citrate
Creatine
Creatine phosphate
Ethanol
Formate
Glucose
Glutamate
Hypotaurine
Isoleucine
Lactate
Leucine
Malonate
Methanol
Myo-Inositol
Phenylalanine
sn-Glycero-3-phosphocholine
Trimethylamine N-oxide
Tyrosine
Valine
-20000
-10000
0

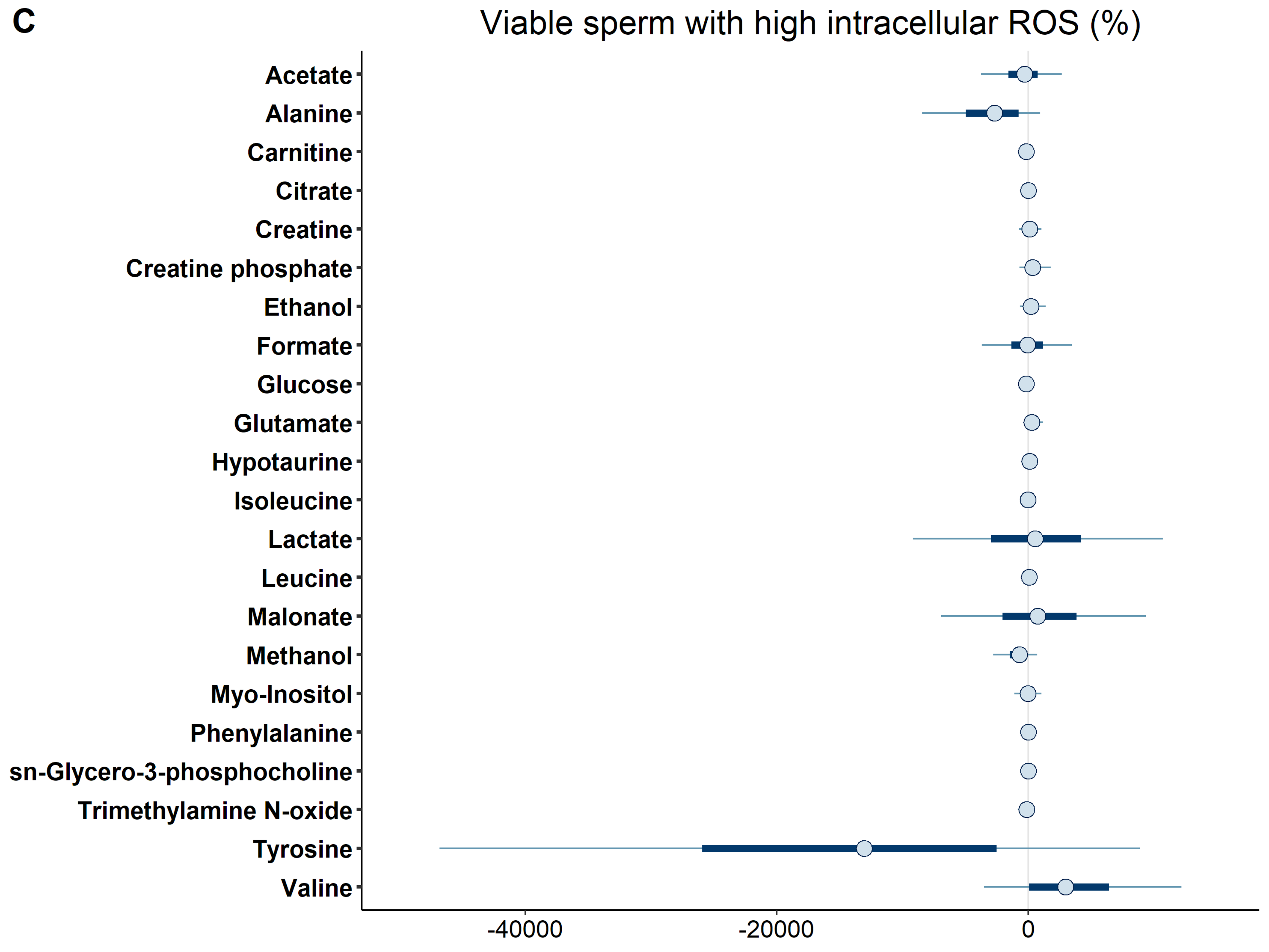
C
Viable sperm with high intracellular ROS (%)
Acetate
Alanine
Carnitine
Citrate
Creatine
Creatine phosphate
Ethanol
Formate
Glucose
Glutamate
Hypotaurine
Isoleucine
Lactate
Leucine
Malonate
Methanol
Myo-Inositol
Phenylalanine
sn-Glycero-3-phosphocholine
Trimethylamine N-oxide
Tyrosine
Valine
-40000
-20000
0

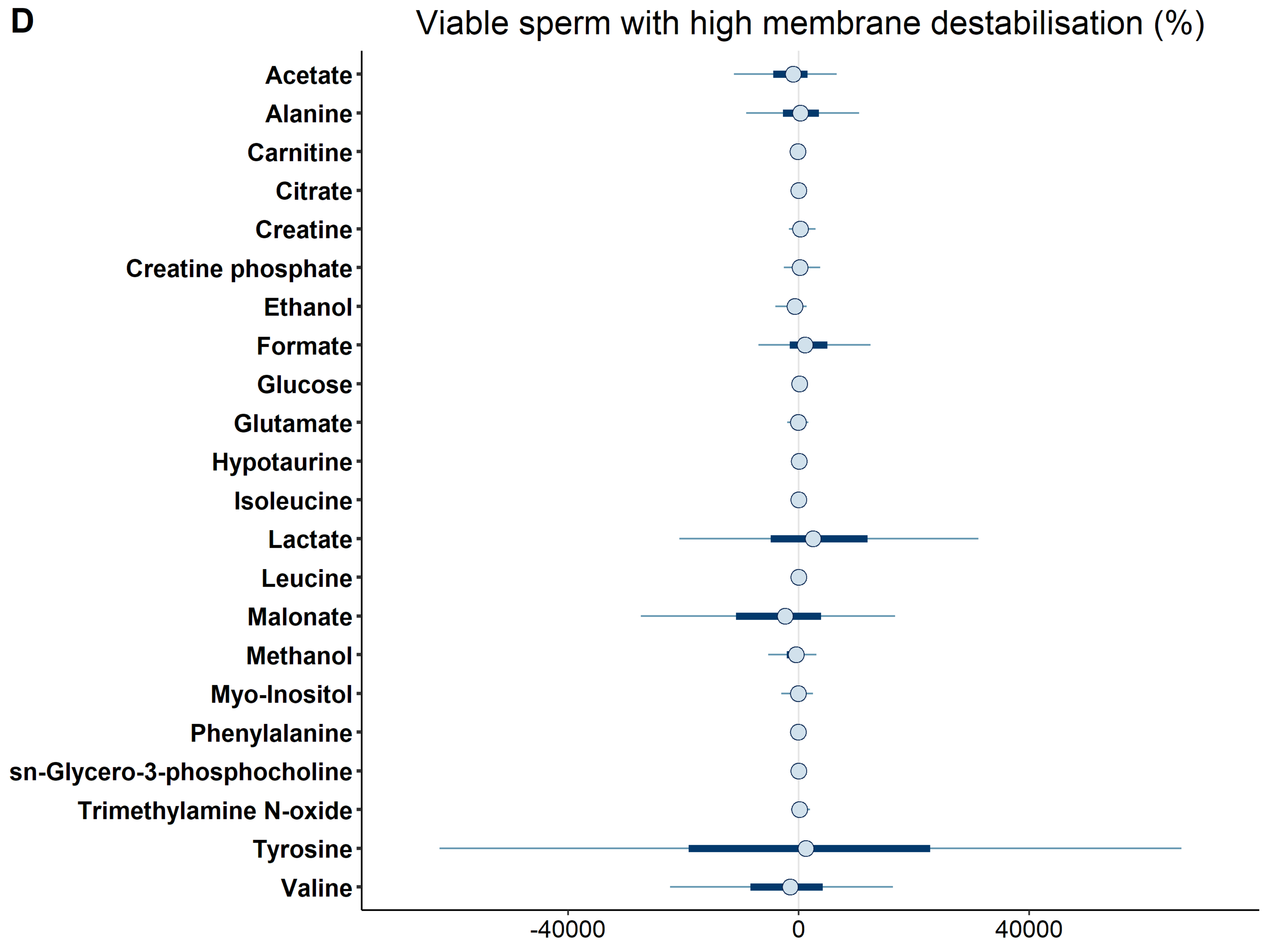
D
Viable sperm with high membrane destabilisation (%)
Acetate
Alanine
Carnitine
Citrate
Creatine
Creatine phosphate
Ethanol
Formate
Glucose
Glutamate
Hypotaurine
Isoleucine
Lactate
Leucine
Malonate
Methanol
Myo-Inositol
Phenylalanine
sn-Glycero-3-phosphocholine
Trimethylamine N-oxide
Tyrosine
Valine
-40000
0
40000

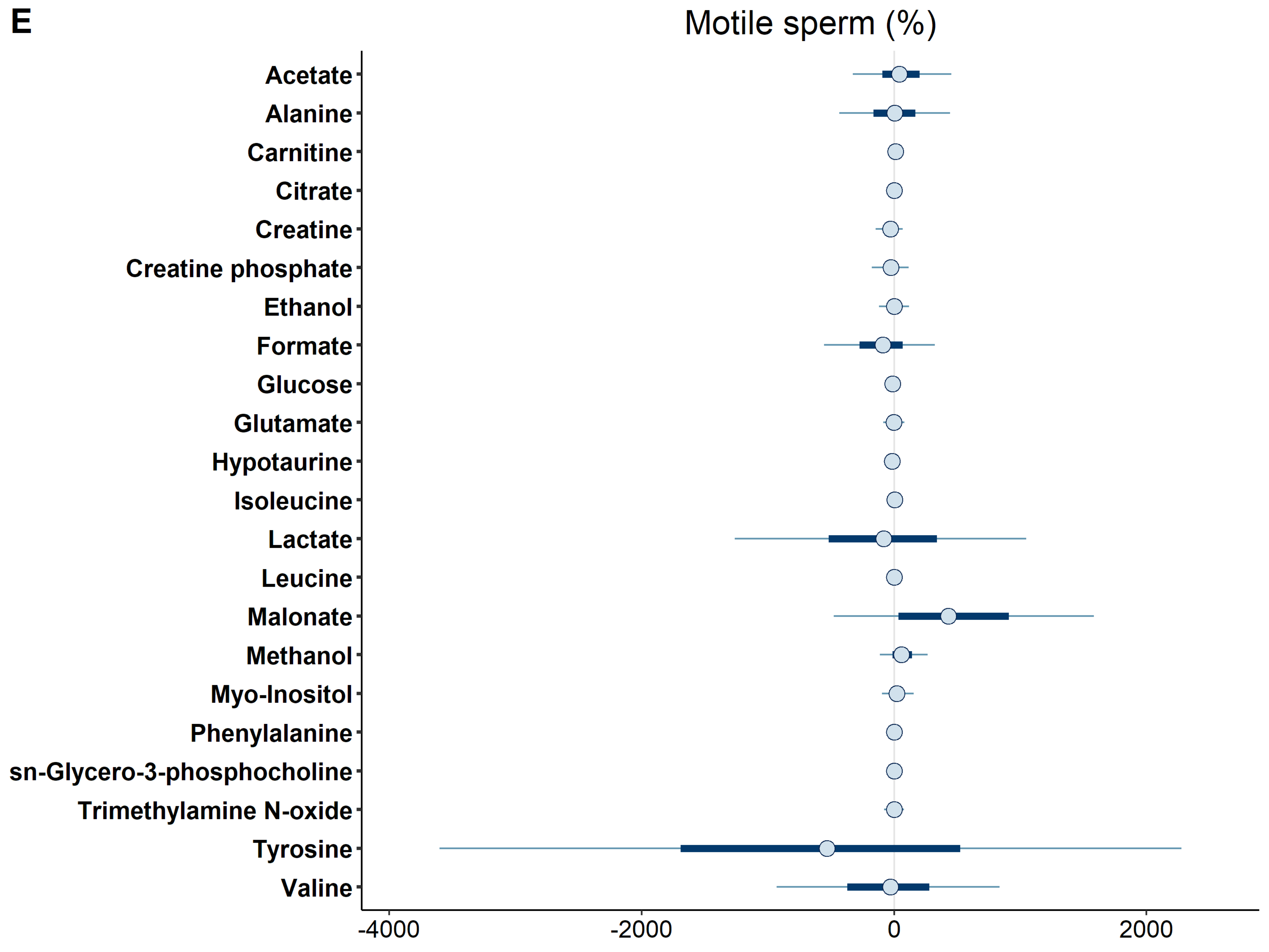

E
Motile sperm (%)
Acetate
Alanine
Carnitine
Citrate
Creatine
Creatine phosphate
Ethanol
Formate
Glucose
Glutamate
Hypotaurine
Isoleucine
Lactate
Leucine
Malonate
Methanol
Myo-Inositol
Phenylalanine
sn-Glycero-3-phosphocholine
Trimethylamine N-oxide
Tyrosine
Valine
-4000
-2000
0
2000

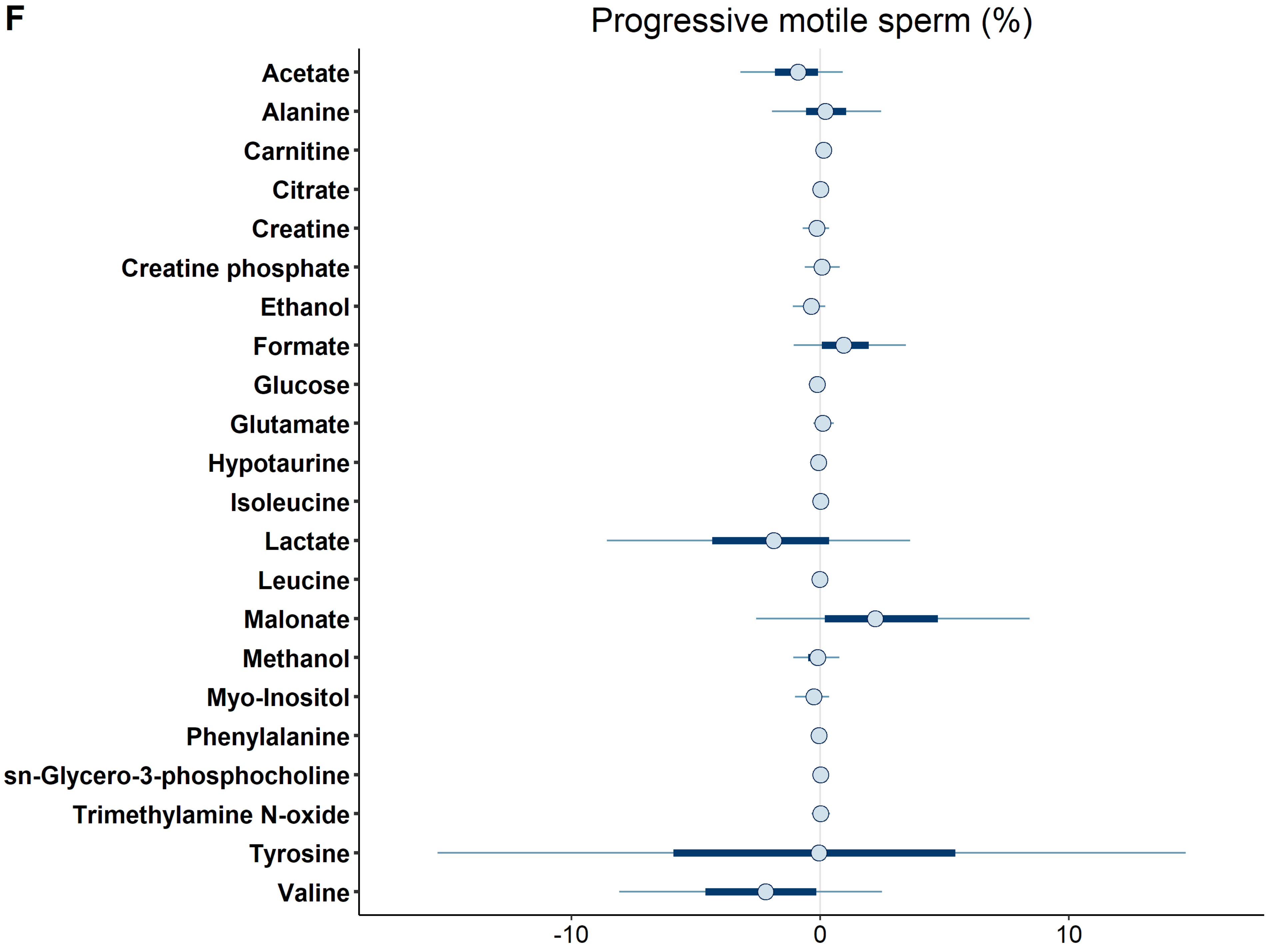

F
Progressive motile sperm (%)
Acetate
Alanine
Carnitine
Citrate
Creatine
Creatine phosphate
Ethanol
Formate
Glucose
Glutamate
Hypotaurine
Isoleucine
Lactate
Leucine
Malonate
Methanol
Myo-Inositol
Phenylalanine
sn-Glycero-3-phosphocholine
Trimethylamine N-oxide
Tyrosine
Valine
-10
0
10

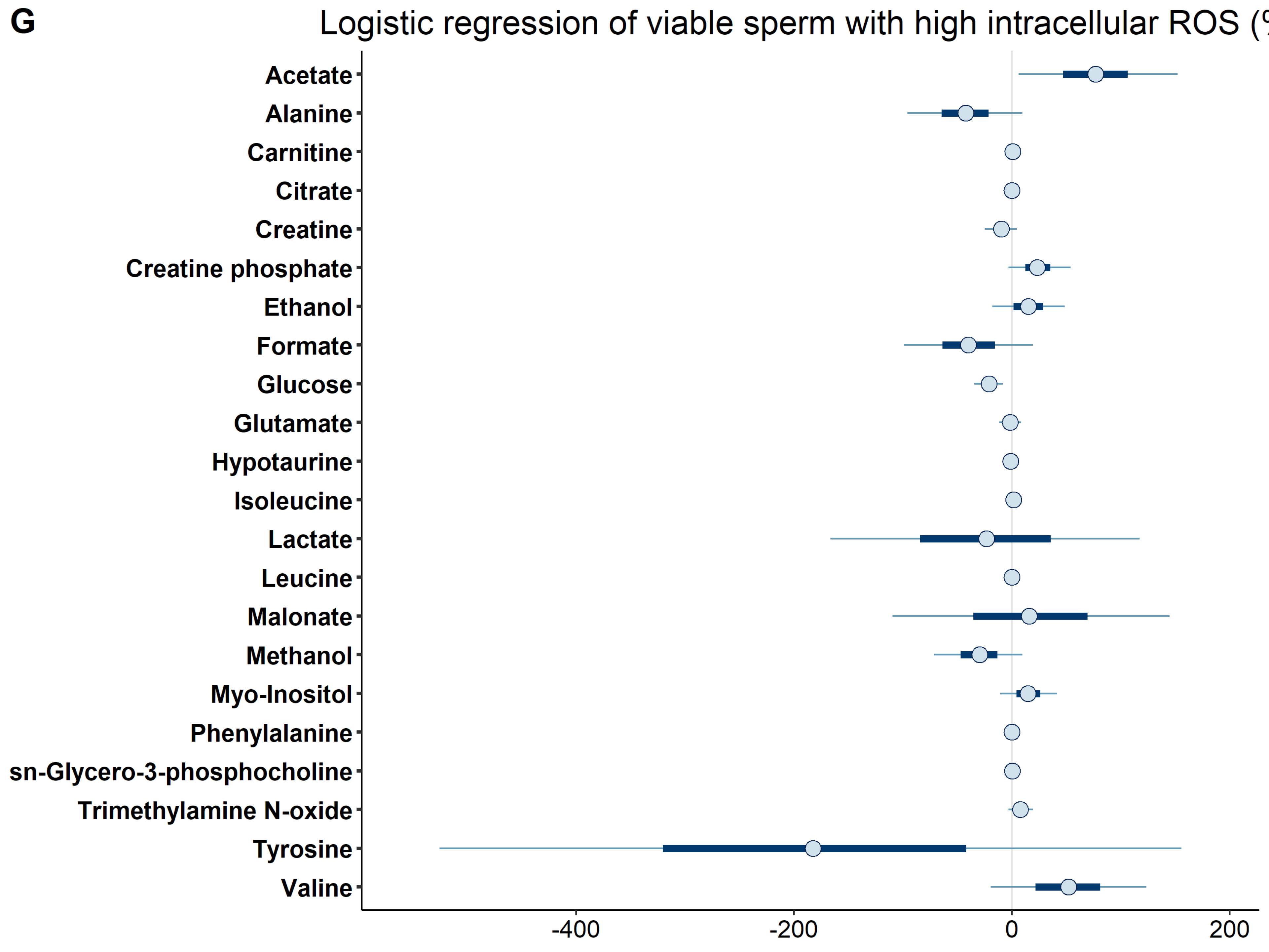
G
Logistic regression of viable sperm with high intracellular ROS (%
Acetate
Alanine
Carnitine
Citrate
Creatine
Creatine phosphate
Ethanol
Formate
Glucose
Glutamate
Hypotaurine
Isoleucine
Lactate
Leucine
Malonate
Methanol
Myo-Inositol
Phenylalanine
sn-Glycero-3-phosphocholine
Trimethylamine N-oxide
Tyrosine
Valine
-400
-200
0
200

H

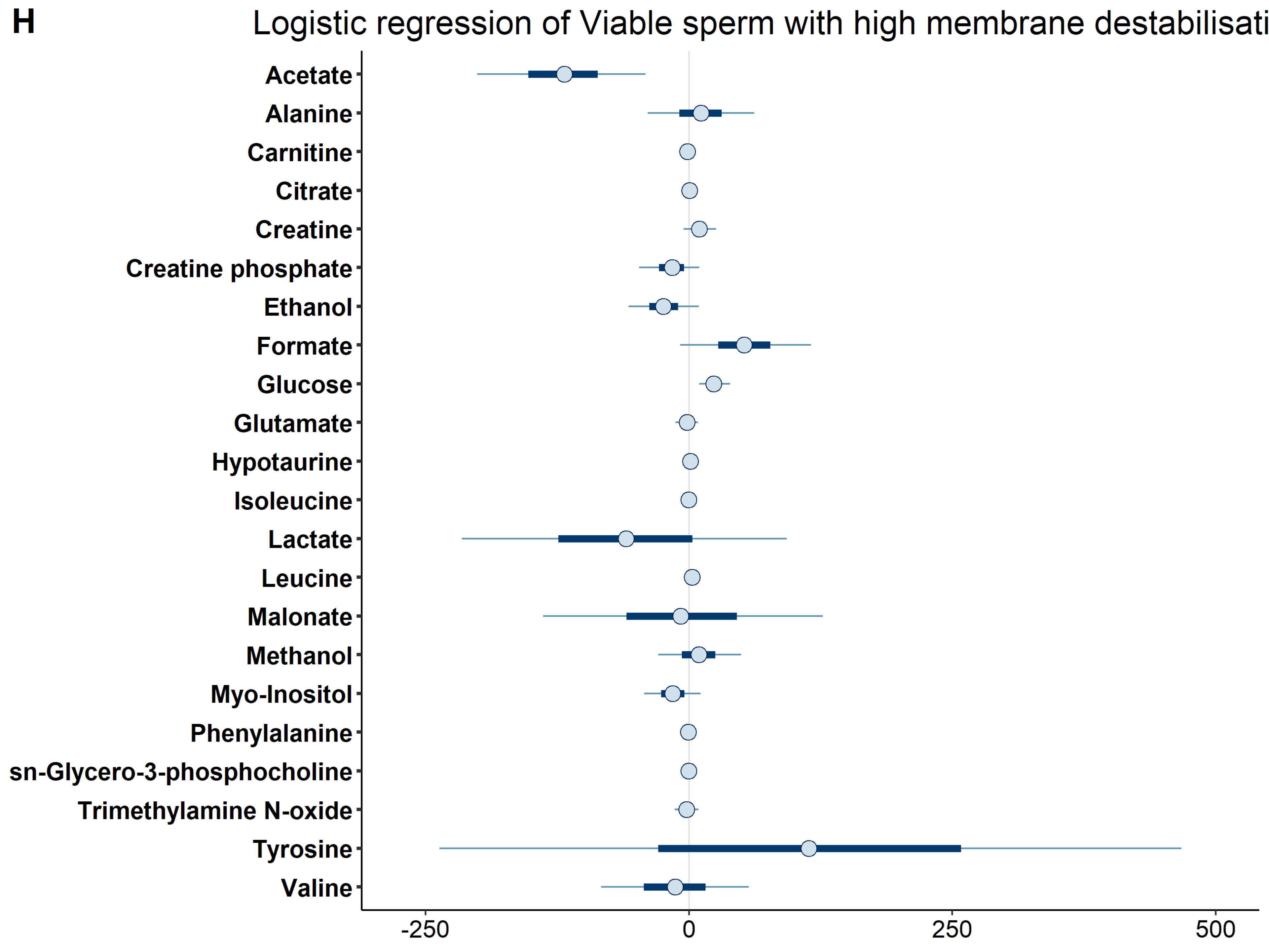
Logistic regression of Viable sperm with high membrane destabilisati
Acetate
Alanine
Carnitine
Citrate
Creatine
Creatine phosphate
Ethanol
Formate
Glucose
Glutamate
Hypotaurine
Isoleucine
Lactate
Leucine
Malonate
Methanol
Myo-Inositol
Phenylalanine
sn-Glycero-3-phosphocholine
Trimethylamine N-oxide
Tyrosine
Valine
-250
0
250
500
