# Supplementary material for: Metabolite Profiling of Pig Seminal Plasma Identifies Potential Biomarkers for Sperm Resilience to Liquid Preservation
Source: Front Cell Dev Biol. 2021 May 28;9:669974. doi: 10.3389/fcell.2021.669974 (PMC8194698; doi:10.3389/fcell.2021.669974)
Supplement: Supplementary Table 1 — Metabolite concentrations (mM) in seminal plasma of Pietrain and Duroc breeds. Significant correlations (P < 0.05) are marked with ∗. [file Data_Sheet_1.docx]

**Supplementary Materials**

**Supplementary Figures (Images 1-15)**

**Supplementary Figure 1 (A-M).** Complete Bayesian multiple linear regression models for all the parameters evaluated immediately after semen collection.

**Supplementary Figure 2.** **(A-F)** Complete Bayesian multiple linear regression models for all the parameters evaluated immediately after semen collection. **(G-H)** Complete Bayesian logistic regression models for percentages of viable sperm with high membrane destabilization, and with high intracellular ROS.

**Supplementary Tables**

**Supplementary Table 1.** Metabolite concentrations (mM) in seminal plasma of Pietrain and Duroc breeds. Significant correlations (*P* < 0.05) are marked with *.

| Metabolite | Pietrain | | | Duroc | | |
| --- | --- | --- | --- | --- | --- | --- |
|  | **Median** | **Quartile 25** | **Quartile 75** | **Median** | **Quartile 25** | **Quartile 75** |
| Acetate | 0.1309 | 0.1217 | 0.1380 | 0.1229 | 0.0970 | 0.1491 |
| Alanine | 0.1781 | 0.1076 | 0.2274 | 0.1189 | 0.0882 | 0.1484 |
| Carnitine | 1.0370 | 0.6533 | 1.2626 | 0.7736 | 0.6110 | 1.0126 |
| Citrate | 7.1768 | 6.0597 | 8.7712 | 5.9533 | 4.4453 | 6.9273 |
| Creatine | 0.3897 | 0.2645 | 0.5209 | 0.3457 | 0.2544 | 0.4654 |
| Creatine phosphate | 0.0667 | 0.0576 | 0.0972 | 0.0480 | 0.0408 | 0.0759 |
| Ethanol | 0.1986 | 0.1726 | 0.2085 | 0.2009 | 0.1589 | 0.2157 |
| Formate | 0.1927 | 0.1846 | 0.2046 | 0.1976 | 0.1787 | 0.2266 |
| Fumarate | 0.0017* | 0.0016 | 0.0021 | 0.0029 | 0.0021 | 0.0048 |
| Glucose | 0.1527 | 0.0947 | 0.2334 | 0.1492 | 0.0739 | 0.2367 |
| Glutamate | 0.6271 | 0.3736 | 0.8469 | 0.4438 | 0.3849 | 0.5235 |
| Hypotaurine | 2.2498 | 1.5999 | 3.5265 | 1.8605 | 1.5945 | 2.3641 |
| Isoleucine | 3.2218 | 2.7607 | 4.5459 | 2.6861 | 2.0976 | 3.2277 |
| Lactate | 0.0506 | 0.0284 | 0.0702 | 0.0486 | 0.0350 | 0.0609 |
| Leucine | 2.3225 | 1.6093 | 2.4359 | 1.7699 | 1.3321 | 1.9681 |
| Malonate | 0.0653 | 0.0469 | 0.0700 | 0.0655 | 0.0457 | 0.0742 |
| Methanol | 0.1136 | 0.0837 | 0.1929 | 0.0994 | 0.0694 | 0.1614 |
| Myo-Inositol | 0.0829 | 0.0442 | 0.1216 | 0.0478 | 0.0200 | 0.0998 |
| Phenylalanine | 4.9352 | 3.6804 | 8.1076 | 4.0131 | 2.9027 | 4.6300 |
| sn-Glycero-3-phosphocholine | 27.6931 | 22.5105 | 31.2527 | 21.4458 | 18.0925 | 27.3980 |
| Trimethylamine N-oxide | 0.2836 | 0.1794 | 0.6347 | 0.2943 | 0.2193 | 0.3913 |
| Tyrosine | 0.0264 | 0.0178 | 0.0324 | 0.0212 | 0.0187 | 0.0285 |
| Valine | 0.1171 | 0.0882 | 0.1323 | 0.1031 | 0.0837 | 0.1454 |

**Supplementary Table 2.** Sperm quality and functionality parameters of pig semen samples (n = 28) assessed immediately after semen collection. SD: Standard Deviation.

| Quality parameter | Mean ± SD |
| --- | --- |
| Viable sperm (%) | 85.29 ± 6.065 |
| Viable sperm with a non-intact acrosome (%) | 1.58 ± 0.922 |
| Viable sperm with high intracellular ROS (%) | 28.89 ± 16.959 |
| Viable sperm with high membrane destabilization (%) | 1.58 ± 0.155 |
| Motile sperm (%) | 78.07 ± 8.671 |
| Progressive motile sperm (%) | 49.25 ± 10.932 |
| Normal morphology (%) | 78.71 ± 12.967 |
| Coiled tails (%) | 0.43 ± 0.790 |
| Folded tails (%) | 6.77 ± 7.709 |
| Acrosome abnormalities (%) | 2.64 ± 4.057 |
| Proximal droplets (%) | 6.05 ± 6.245 |
| Distal droplets (%) | 4.64 ± 6.225 |
| Abnormal head size of shape (%) | 0.75 ± 1.456 |

**Supplementary Table 3.** Sperm quality loss of pig semen samples (n = 28) after 72 h of liquid storage at 17 ºC. SD: Standard Deviation.

| Quality parameter | Mean ± SD |
| --- | --- |
| Viable sperm (%) | -1.67 ± 3.466 |
| Viable sperm with non-intact acrosome (%) | 47.54 ± 73.694 |
| Viable sperm with high intracellular ROS (%) | 143.33 ± 167.136 |
| Viable sperm with high membrane destabilization (%) | 345.23 ± 519.590 |
| Motile sperm (%) | -10.91 ± 19.180 |
| Progressive motile sperm (%) | 0.744 ± 0.094 |
